# Effect and Response of *Quercus ilex* subsp. *ballota* [Desf.] Samp. Seedlings From Three Contrasting Andalusian Populations to Individual and Combined *Phytophthora cinnamomi* and Drought Stresses

**DOI:** 10.3389/fpls.2021.722802

**Published:** 2021-08-19

**Authors:** Bonoso San-Eufrasio, María Ángeles Castillejo, Mónica Labella-Ortega, Francisco J. Ruiz-Gómez, Rafael M. Navarro-Cerrillo, Marta Tienda-Parrilla, Jesús V. Jorrín-Novo, María-Dolores Rey

**Affiliations:** ^1^Agroforestry and Plant Biochemistry, Proteomics and Systems Biology, Department of Biochemistry and Molecular Biology, University of Córdoba, Córdoba, Spain; ^2^Evaluation and Restoration of Agronomic and Forest Systems ERSAF, Department of Forest Engineering, University of Córdoba, Córdoba, Spain

**Keywords:** holm oak, decline syndrome, climate change, combined stress, proteomics, molecular markers

## Abstract

*Quercus ilex* L. is the dominant species in the Mediterranean forest and agrosilvopastoral ecosystem “*dehesa*.” Currently, this forest species is threatened by natural and anthropogenic agents, especially by the decline syndrome, which is caused by *Phytophthora cinnamomi* and drought periods. Although the morphological and physiological responses of *Q. ilex* to combined stress (*P. cinnamomi* and drought) have been examined already, little is known at the molecular level. In this study, we studied the effect and response of 8-month seedlings from three contrasting Andalusian populations (Seville [Se], Granada [Gr], and Almeria [Al]) to the individual and combined stresses of *P. cinnamomi* and drought from morphological, physiological, biochemical, and proteomics data. Whereas, seedling damage (leaf chlorosis and necrosis) and mortality were greater under the combined stresses in the three populations, the effect of each individual stress was population-dependent. Resilient individuals were found in all the populations at different percentages. The decrease in leaf chlorophyll fluorescence, photosynthetic activity, and stomatal conductance observed in undamaged seedlings was greater in the presence of both stresses, the three populations responding similarly to drought and *P. cinnamomi*. Biochemical and proteomic analyses of undamaged seedlings from the two most markedly contrasting populations (Se and Al) revealed the absence of significant differences in the contents in photosynthetic pigments, amino acids, and phenolics among treatments. The Se and Al populations exhibited changes in protein profile in response to the different treatments, with 83 variable proteins in the former population and 223 in the latter. Variable proteins belonged to 16 different functional groups, the best represented among which were protein folding, sorting and degradation, carbohydrate, amino acid, and secondary metabolism, photosynthesis, and ROS scavenging. While photosynthetic proteins were mainly downaccumulated, those of stress-responsive were upaccumulated. Although no treatment-specific response was observed in any functional group, differences in abundance were especially marked under the combined stresses. The following variable proteins are proposed as putative markers for resilience in *Q. ilex*, namely, aldehyde dehydrogenase, glucose-6-phosphate isomerase, 50S ribosomal protein L5, and α-1,4-glucan-protein synthase [UDP-forming].

## Introduction

Holm oak (*Quercus ilex* L.) is the dominant species in Mediterranean basin forests and also in the long-established agrosilvopastoral oak open woodlands called *dehesas* in Spain and *montados* in Portugal (Ruiz de la Torre, 2006; Camilo-Alves et al., 2013). This species possesses a high environmental and ecological importance (Guzmán-Álvarez, 2016). It adapts well to arid and semiarid regions, where it plays a key biological role against desertification (Quero et al., 2006). In recent decades, however, increasing tree defoliation and mortality in large areas of the western Iberian Peninsula are endangering holm oak forests along the Mediterranean basin (Brasier, 1996; Jung et al., 2000; Natalini et al., 2016; Sánchez-Cuesta et al., 2021). Tree mortality is associated with both natural and anthropogenic factors such as overexploitation and poor regeneration or livestock management, and also with the severe effect of external biotic and abiotic factors such as attack by soilborne pathogens, extreme temperatures, heavy rainfall episodes, and extended drought periods, which in combination result in the so-called “oak decline syndrome” (Brasier, 1992; Camilo-Alves et al., 2013; Corral-Ribera et al., 2018; Surová et al., 2018).

Holm oak decline is a complex syndrome usually triggered by extreme climate events such as drought and high temperatures or invasive pathogens such as oomycetes (Sánchez et al., 2004; Sghaier-Hammami et al., 2013; Valero-Galván et al., 2013; Ruiz-Gómez et al., 2018). However, there is solid evidence that drought and root rot by the effect of *Phytophthora cinnamomi* Rands. are the two main factors triggering strong tree death episodes (Brasier, 1996; Sánchez et al., 2004; Corcobado et al., 2014, 2017; Ruiz-Gómez et al., 2018, 2019; Gea-Izquierdo et al., 2021). *P. cinnamomi* is one of the worst invasive alien pathogens around the world (Burgess et al., 2016), and its spectrum of hosts includes more than 5,000 different species (Hardham, 2005). This oomycete is heterothallic (i.e., it has two different mating types, of which only type A2 is present in the Iberian Peninsula). The pathogen reproduces asexually by sporulating motile zoospores that can be carried by soil water to find new roots and spread easily under the typical conditions of Mediterranean climate (*viz*., short episodes of heavy rainfall, intermittent flooding, and heavy runoff) (Brasier, 1992, 1996; Hardham, 2005). The geographical origin of *P. cinnamomi* is not clearly established. It is an introduced species in Europe, which probably comes from Taiwan or the islands of Papua New Guinea in the south-western Pacific Ocean (Sena et al., 2018). Although in some places *P. cinnamomi* seems to be a relatively new threat, in other parts it has been established for many decades. In Europe, *P. cinnamomi* was identified in chestnuts causing ink disease in the last of the 19th century (Brasier, 1996), and in the Iberian Peninsula, since the end of the last century *P. cinnamomi* has been associated with oak declining (Brasier et al., 1993).

The previous studies have shown drought to increase the susceptibility of *Q. ilex* seedlings to *P. cinnamomi* root rot (Corcobado et al., 2014, 2017; Ruiz-Gómez et al., 2018). This finding is supported by the fact that plants are weakened under environmental cues (Agrios, 2005; Desprez-Loustau et al., 2006). The combined effects of biotic and abiotic stresses on *Q. ilex* seedlings have so far been studied in phenotypic, physiological, and biochemical terms (Corcobado et al., 2014; Ruiz-Gómez et al., 2018, Colangelo et al., 2018). Thus, the presence of *P. cinnamomi* is known to trigger unspecific defense responses, such as the accumulation of phenolics, thickening of cell walls, and accumulation of callose (Ruiz Gómez et al., 2014; Redondo et al., 2015). Under drought, the presence of *P. cinnamomi* results in substantial changes in biomass allocation such as a decrease in root biomass and also in physiological activity-related parameters such as CO_2_ assimilation, stomatal conductance, and leaf chlorophyll fluorescence (Ruiz-Gómez et al., 2018). These changes must, no doubt, reflect at the molecular level, but how it does remains largely unknown to date. Recently, Ruiz-Gómez et al. (2018) examined changes in a *Q. ilex* population in Arenas del Rey (Granada, Andalusia, Spain) under stress from both *P. cinnamomi* and drought. However, the high interpopulation and intrapopulation variability of this species (San-Eufrasio et al., 2020) requires comparing various populations in order to better elucidate the response of holm oak to the conditions causing the decline syndrome. Also, molecular studies using the most recent tools available for this purpose could be useful to gain further insight into variability in this species and also to help identify key genes and gene products involved in the response to the syndrome (Rey et al., 2019).

In this study, we studied the effect of exposure of *Q. ilex* to drought and attack by a pathogen (*P. cinnamomi*) individually and in combination from a physiological, biochemical, and proteomic perspective. For this purpose, we examined the response to and tolerance of *P. cinnamomi* and drought in three contrasting Andalusian *Q. ilex* populations (Seville [Se], Granada [Gr], and Almeria [Al]). Elucidating the molecular mechanisms behind resilience to both stresses in *Q. ilex* from physiological and molecular data allowed us to put forward several putative gene markers for use in breeding actions in the framework of conservation and afforestation programs.

## Materials and Methods

### Plant Material

Acorns were collected by staff of the Department of Forestry Engineering of the University of Cordoba from three different *Q. ilex* populations in Andalusia, namely, Almaden de la Plata, Se; Gr); and Al) (Table 1; Supplementary Figure 1).

**Table 1:** Location and environmental features of the three Andalusian *Quercus ilex* populations.

	**Location**	**Altitude**	**Coordinates**	***T*_**max**_**	***T*_**min**_**	***P***
More Eastern	Sierra María (Al)	890	37°42′ N 2°07′ W	18.0	5.8	411.0
↓	Arenas del Rey (Gr)	892	36°57′ N 3°54′ W	24.7	11.5	489.3
More Western	Almaden de la Plata (Se)	482	37°52′ N 6°05′ W	26.4	9.5	722.1

*Altitude (meters above sea level, MASL), ETRS89 coordinates, the average temperature of the coldest month (T_min_, °C), the average temperature of the warmest month (T_max_, °C), and average annual rainfall (P, mm) (Navarro-Cerrillo et al., 2018)*.

Healthy acorns were selected after surface cleaning with 5% HCl and suspension in water, with floating acorns being discarded. In January 2019, acorns were pregerminated in a wet bed and sown in black plastic pots (3 L, 14.5 × 14.5 × 22 cm) containing perlite from Gramoflor GmbH and Co. (Vechta, Germany). Pots were placed in a temperature-controlled greenhouse at a mean temperature of 25°C, 60 ± 10% relative humidity, PPFD of 900 μmol m^−2^ s ^−1^, and natural photoperiod (11/13 h, light/dark) located in Cordoba (Andalusia, Southern Spain; 37°54′46″ N, 4°43′15″ O). During the experiment, the maximum temperature did not exceed of 39°C and did not fall below 13°C. The experiment was started in October 2019, when seedlings were ~15 cm tall. Seedlings were irrigated every 2 days with tap water (200 ml) and once a week with Hoagland nutrient solution up to the start of the experiment (Hoagland and Arnon, 1950).

### Experimental Design and Inoculation

The experimental design (Supplementary Figure 2) encompassed four different treatments, namely, (1) irrigation to field capacity in the absence of *P. cinnamomi* (control treatment); (2) no irrigation and no *P. cinnamomi* (drought treatment); (3) irrigation and *P. cinnamomi* inoculation (inoculation treatment); and (4) *P. cinnamomi* inoculation and no irrigation (combined treatment). The treatments were performed as described by Ruiz-Gómez et al. (2018) and Sghaier-Hammami et al. (2013). The experiment was conducted according to a completely randomized design with 20 seedlings per treatment (80 seedlings per population) and a duration of 35 days.

*Phytophthora cinnamomi* (P90), previously isolated from *Q. ilex* roots in Puebla de Guzmán (Huelva, Andalusia, Spain), was reactivated by using root cuts inoculated in a PARPBH selective medium containing piramicin, amplicilin, rifamycin, pentachloronitrobenzene, benomyl, and hymexazol (Jeffers, 1986; Ruiz-Gómez et al., 2018). The inoculation protocol used was that of Ruiz-Gómez et al. (2018) except that the root system was brought into contact with the pathogen by immersion in Carror–Agar (CA) liquid inoculum (Sghaier-Hammami et al., 2013) at a concentration of 39 chlamydospores/μL (Ruiz-Gómez et al., 2018). Control seedlings were also immersed in CA liquid inoculum but containing no *P. cinnamomi*. After inoculation, seedlings were transplanted into pots filled with fresh perlite. All pots were flooded for 48 h, excess water being removed before the experiment. In the drought treatment, water was withheld according to San-Eufrasio et al. (2020). Control and inoculated seedlings were irrigated to field capacity throughout. The presence of *P. cinnamomi* in the root system was checked on days 19 and 32 by using fine roots from each seedling as described by Ruiz-Gómez et al. (2018). Briefly, three pieces of fine roots per seedling (<2 mm thick, ~1 cm long) were randomly selected and immersed in 70% ethanol, washed in sterilized–deionized water, and placed in 9-cm Petri dishes containing PARPBH selective medium. The pathogen was identified morphologically by conventional light microscopy (Erwin and Ribeiro, 1996).

### Perlite Water Content and Matric Potential

Both the perlite water content (PWC, %) and the matric potential (Ψ_m_, kPa) were estimated according to San-Eufrasio et al. (2020) throughout the experiment. The former parameter was calculated as follows:

$$\begin{array}{l} {\text{PWC}}_{t}\left(\%\right)=\left[{\text{PWW}}_{t}-\left({\text{PDW}/\text{PWW}}_{0}\right)-\text{PDW}\right]\times 100 \end{array}$$

where PWW denotes pot wet weight; PDW pot dry weight; and *t* time, in days, with 0 corresponding to the initial and maximum values.

### Damage Symptoms and Seedling Mortality

Damage symptoms (*viz*., leaf chlorosis and wilting) resulting from the presence of *P. cinnamomi* and/or drought were evaluated visually in all seedlings and registered by taking photographs of all seedlings with a digital camera. When all leaves exhibited severe drought symptoms (*viz*., a dry-yellow appearance throughout) and quantum yield of photosystem II (Qy) was near 0, the number of dead seedlings was also recorded.

### Physiological Measurements

Relative leaf water content (RLWC) was calculated on day 32 from fresh (FW), turgid (TW), and dry (DW) weights, as previously described by San-Eufrasio et al. (2020). RLWC (%) was calculated as [(FW–DW)/(TW – DW)] × 100. The Qy was measured with a FluorPen FP100 portable leaf fluorimeter from Photon Systems Instruments (Drasiv, Czech Republic) at 3-day intervals throughout the experiment (San-Eufrasio et al., 2020). Measurements were always performed on the same three youngest fully expanded leaves in each seedling, using three seedlings per treatment per population (36 seedlings per measurement) throughout the experiment. All measurements were performed in the early morning when the leaves were adapted to darkness throughout the night (dark-adapted leaves) according to Strasser et al. (2000). Net photosynthesis (A, μmol CO_2_ m^−2^ s^−1^), stomatal conductance (Gs, mol H_2_O m^−2^ s^−1^), and $F_{\text{v}}^{\prime }$/$F_{\text{m}}^{\prime }$ (maximal Qy photochemistry for the light-adapted state, arbitrary units) were measured once a week on a light-adapted fully expanded leaf in five seedlings per treatment per population, using a portable IR CO_2_ gas analyzer (IRGA) equipped with a light source integrated in a leaf chamber fluorometer and CO_2_ injector system (LiCor Li6400XT, Li-Cor, Inc.; Lincoln, NE, USA). The measurements were taken fixing a CO_2_ concentration of 400 ppm and PPFD of 1,000 μmol (photons) m^−2^ s^−1^. Relative humidity was setup to environmental RH (15.5+- 0.2 mmol H_2_O mol air^−1^), and the length of measurements was controlled by the saturation of the A and G curves, establishing a minimum run time of 30 s. All measurements were performed between 11:00 and 14:30 UTC (Universal Time Coordinates).

### Photosynthetic Pigment and Metabolite Contents

Photosynthetic pigments and metabolites were quantified in asymptomatic leaves from the Se and Al populations. Measurements were performed on day 32, when leaf chlorophyll fluorescence had decreased by 20, 30, and 35% in the drought, inoculation, and combined treatment, respectively, relative to the control seedlings (López-Hidalgo et al., 2021). Three biological replicates per treatment per population were used for this purpose. Leaves from each seedling were collected, washed with distilled water, and immersed in liquid nitrogen prior to grinding in a precooled mortar. Leaf tissue (30–50 mg dry weight) was extracted with 80% (v/v) ethanol, the crude extract being centrifuged, and the resulting supernatant collected to quantify photosynthetic pigments (chlorophyll a and b, and carotenoids) and metabolites (total free amino acids, soluble sugars, total phenolics, and total flavonoids) on an Evolution 201 UV–Vis spectrophotometer from Thermo Fischer Scientific (Waltham, MA, USA) (López-Hidalgo et al., 2021). The resulting pellet was extracted with perchloric acid to quantify starch (López-Hidalgo et al., 2021) by measuring the absorbance at 595 nm of the supernatant against a hydrolyzed starch standard (Viles and Silverman, 1949; Rose et al., 1991). The absorbance of the supernatants containing chlorophyll a, chlorophyll b, and carotenoids was recorded at 664, 649, and 470 nm, respectively, and was used to calculate the respective contents, all in micrograms per milliliter, as follows: chlorophyll a = 13.36·*A*_664_-5.19·*A*_649_; chlorophyll b = 27.43·*A*_649_ – 8.12·*A*_664_; carotenoids = [(1000·*A*_470_ – 2.13·chlorophyll a – 97.63·chlorophyll b)/209] (Lichtenthaler, 1987). The supernatants were used to calculate the content of free amino acids, soluble sugars, total phenolics, and total flavonoids as follows: For total free amino acids, the crude extract was mixed thoroughly with a commercial (2:1 v/v) ninhydrin reagent (Starcher, 2001) and the absorbance was measured at 450 nm using a standard of (1:1) proline–glycine; for soluble sugars, the crude extract was mixed thoroughly with (1:16 v/v) anthrone reagent (Thayumanavan and Sadasivam, 1984) and the absorbance was measured at 595 nm using a standard of (1:1) glucose; for total phenolics, the supernatants were mixed thoroughly with 10% (1:2 v/v) Folin–Ciocalteu reagent followed by the addition of (3:8 v/v) sodium carbonate (Viles and Silverman, 1949) and the absorbance measured at 720 nm using a standard of (1:1) gallic acid; and for flavonoids, the crude extract was mixed thoroughly with 10% (10:1) (v/v) aluminum chloride, 1 M potassium acetate, and (22:35 v/v) methanol (Mammen and Daniel, 2012), with the absorbance measured at 415 nm by using a standard of (1:1) quercetin.

### Protein Extraction and Quantification

Proteomic runs were also performed on the Se and Al populations. Protein extracts were obtained from 300 mg of fresh leaf tissue from asymptomatic seedlings using the TCA/acetone–phenol protocol (Wang et al., 2006). Proteins were extracted from three independent biological replicates under each set of experimental conditions and dissolved in a solution containing 7 M urea, 2 M thiourea, 4% (w/v) CHAPS {3-[(3-cholamidopropyl)dimethylammonium]-1-propanesulphonate}, 0.5% (w/v) Triton X-100, and 100 mM DTT. Protein contents were quantified with the Bradford method using bovine serum albumin (BSA) as the standard (Bradford, 1976).

### Gel-Based Proteomic Analysis (SDS-PAGE)

The proteins extracted from each sample (80 μg of BSA protein equivalent) were separated by sodium dodecyl sulfate polyacrylamide gel electrophoresis (SDS-PAGE) on 12% polyacrylamide gel (Laemmli, 1970), using the Protean II-XL (20 × 20 cm) system from Bio-Rad (Hercules, CA, USA) with a voltage run of 80 V until the dye reached the bottom of the gel. Gels were stained with Coomassie Brilliant Blue R-250 (Neuhoff et al., 1988), and images were acquired with a GS-900 Calibrated Densitometer from Bio-Rad. Images were analyzed with the software ImageLab^TM^ 5.2.1, also from Bio-Rad, bands being automatically detected and confirmed by visual inspection. The optical density (OD) of each band was normalized against the combination of all bands for each sample. Band molecular masses were calculated by comparing mobilities with those of protein standard markers (SDS Molecular weight standards, Broad range, Bio-Rad).

### Shotgun Proteomic Analysis (LC–MS/MS)

Extracted proteins (80 μg) were subjected to SDS-PAGE in a 12% polyacrylamide gel Mini-PROTEAN 8.6 × 6.7 cm^2^ cell from Bio-Rad. The gel was run at 80 V and stopped when Bromophenol Blue had advanced 0.5 cm into the resolving gel. The gel was stained with Coomassie Brilliant Blue R-250. The resulting unique band was removed with a scalpel, cut into pieces smaller than 1 mm3, and transferred individually to 1.5 ml tubes for digestion with 12.5 ng μL^−1^ sequencing-grade trypsin from Promega (Madison, WI, USA) (Castillejo et al., 2015). Peptides were desalinated by passage through a C18 resin microcolumn from Scharlau (Barcelona, Spain), eluted with 70% acetonitrile (AcN) containing 0.1% trifluoroacetic acid, and dried in a SpeedVac apparatus. The resulting peptides were resuspended in 4% (v/v) AcN containing 0.25% (v/v) formic acid (FA) (López-Hidalgo et al., 2018; Romero-Rodríguez et al., 2019). Peptides were charged to 0.4 μg/μl by injection into a one-dimensional nanoflow [i.e., liquid chromatography with tandem mass spectrometry (LC–MS/MS system)] from Thermo Fisher Scientific (Gómez-Gálvez et al., 2020). A monolithic C18 Acclaim PepMap column 15 cm long × 75 μm inner diameter, also from Thermo Fisher Scientific, was used. Peptides were separated at 40°C in all runs. Solvent A contained 0.1% FA, and solvent B consisted of 80% AcN containing 0.1% FA. Samples were separated by using a gradient from 95% solvent A to 80% solvent B at a controlled flow rate of 400 nL/min for 120 min. LC was coupled to MS *via* a nanoelectrospray ionization source. MS analyses were carried out on a trihybrid Thermo Orbitrap Fusion mass spectrometer from Thermo Scientific operated in the positive ion mode. The specific settings used in the LC–MS/MS analysis are described elsewhere (Castillejo et al., 2020).

Raw data were processed with the software MaxQuant (https://www.maxquant.org/). MS2 spectra were searched by using the Andromeda engine against the FASTA *Quercus*_Database obtained from the translation of *Q. ilex* transcriptome (Guerrero-Sanchez et al., 2017, 2019). Trypsin was set as the proteolytic enzyme, and a maximum of two missed cleavages were used in all the searches. Precursor mass tolerance was set at 10 ppm, fragment ion mass tolerance at 0.1 Da, and charge states at +2 or greater. Peptides were classified into proteins according to the law of parsimony and filtered to a 1% false discovery rate (FDR). Identification confidence was set to a minimum score of 2, proteins with two or more peptides matched at least 15% of sequence coverage. Proteins were quantified in relative terms from the peak areas for precursor ions (the average of the three strongest peptide ion signals) from the identified peptides. Then, they were categorized by function from their FASTA sequences using the software Mercator v.3.6 (MapMan) (Lohse et al., 2014; http://www.plabipd.de/portal/mercator-sequence-annotation/, accessed January 2021). Uncharacterized proteins were subjected to gene ontology (GO) enrichment (http://pantherdb.org/, accessed March 2021). MS proteomics raw data were deposited with dataset identifier PXD025704 in the ProteomeXchange Consortium *via* the PRIDE partner repository (Perez-Riverol et al., 2019).

### Statistical Analyses

The effects of inoculation with *P. cinnamomi* and drought on *Q. ilex* were assessed by using the Kaplan–Meier model, which considers both seedling longevity and status (dead or alive) at the final assessment of survival (Esker et al., 2006; Vivas et al., 2012). This model was used in the previous studies of tree seedling survival (Solla et al., 2011) and provides an effective tool for identifying survival patterns between treatments where cumulative hazards over time (i.e., hazard functions) are generally proportional. The process involved calculating the area under the Qy curve. Levene's test was previously used to confirm homoscedasticity in the physiological and biochemical variables. Then, the data were subjected to ANOVA at *p* ≤ 0.05, which means being separated with the LSD *post hoc* test at *p* ≤ 0.05. One-way ANOVA was used to assess the effect of the individual and combined treatments on RLWC; two-way ANOVA to assess the influence of population and treatment as factors in Qy, biochemical parameters, and the total content in proteins as measured with the Bradford method; and three-way ANOVA to evaluate the effects of population, treatment, and time as factors on physiological parameters as determined by IRGA. Physiological data were previously checked for compliance with the normality and variance homoscedasticity conditions (Rohlf and Sokal, 1995). When the latter were not fulfilled, the Kruskal–Wallis test was used. Some conventional biochemical data were transformed for easier handling. Thus, the sugar contents were used in inverted, and the IRGA parameters ($F_{\text{v}}^{\prime }$/$F_{\text{m}}^{\prime }$) were converted into logarithms for A and squared for Gs and Tr.

The ANOVA analyses were carried out with the software Statistix v. 10. A random experimental design with drought and inoculation as main factors was used. Significant (*p* ≤ 0.05) one-way interactions were subjected to multiple comparisons by least square analysis of means. The significance of pairwise comparisons was determined by using Tukey's test at α = 0.05 (Rohlf and Sokal, 1995). The effect of treatments on measured variables was assessed for significance at the 0.05 confidence level. SDS-PAGE band intensities were expressed in relative form by division into the combined intensity of all bands identified in a replicate. Responses were evaluated with provision for repetitiveness between replicates. Significant differences (*p* ≤ 0.05) between bands for different treatments and populations were identified by one-way ANOVA. A PCA was performed with RStudio v. 1.3.1093. Changes in protein abundance were assessed on a heat map using the heatmaply package (Galili et al., 2018) for RStudio v. 4.0.3.

## Results

### Perlite Water Content, Matric Potential, and Plant Infection

Perlite water contents on day 32 ranged from 57% in watered seedlings (control and inoculation treatments) to 12% in droughted ones (drought and combined treatments). These values corresponded to a matric potential of −15 and −43 kPa, respectively. Seedling infection was evaluated in fine roots from each seedling on days 19 and 32. The presence of *P. cinnamomi* in the roots from the inoculated and combined treatments was identified morphologically (see Supplementary Figure 3).

### Damage Symptoms and Mortality Rate

**Figure 1:**
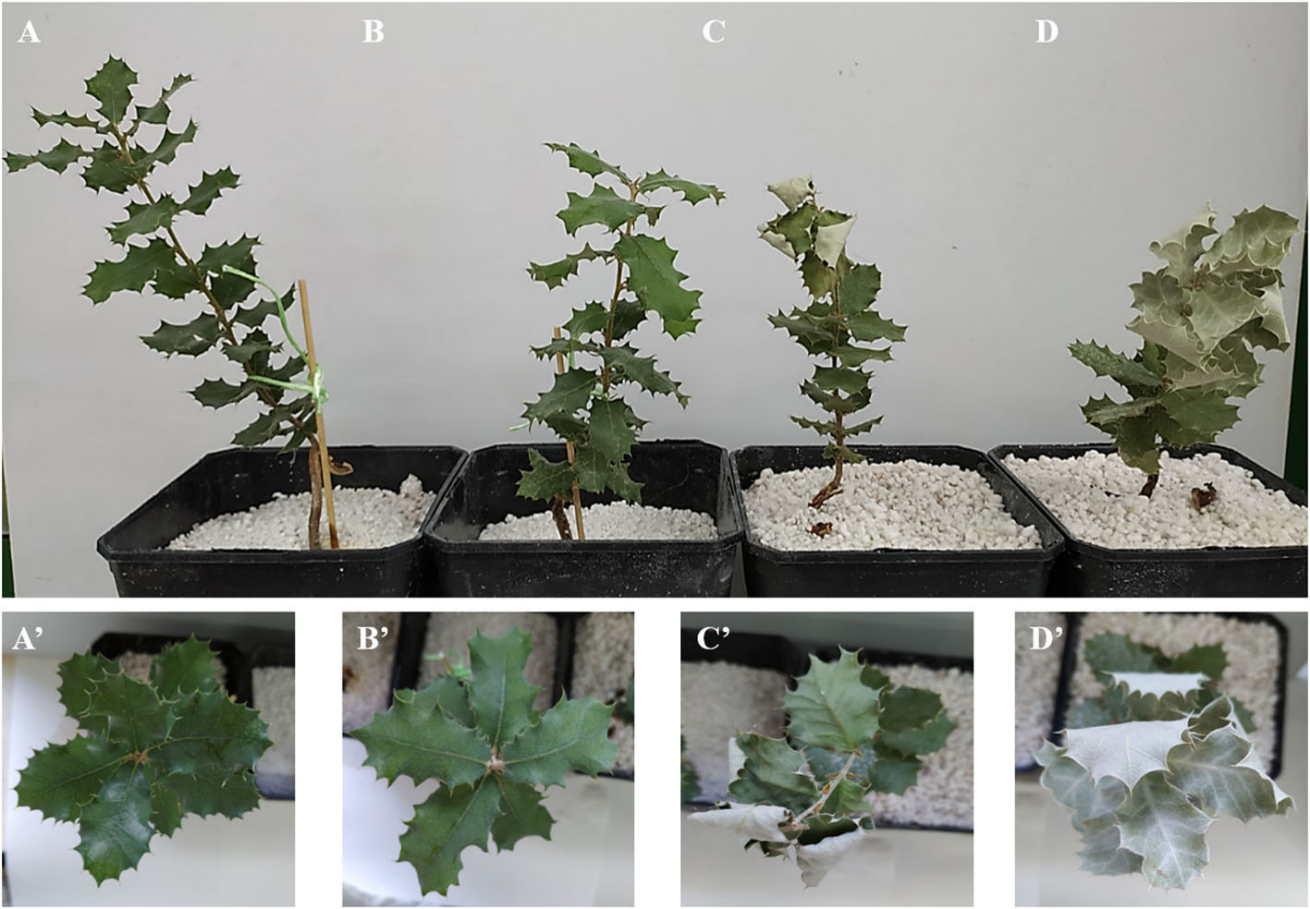
Visually identified damage in *Q. ilex* seedlings from the Seville population Treatments: control (**A,A**'), drought (**B,B**'), *P. cinnamomi* (**C,C**'), and drought × *P. cinnamomi* (**D,D**').

**Figure 2:**
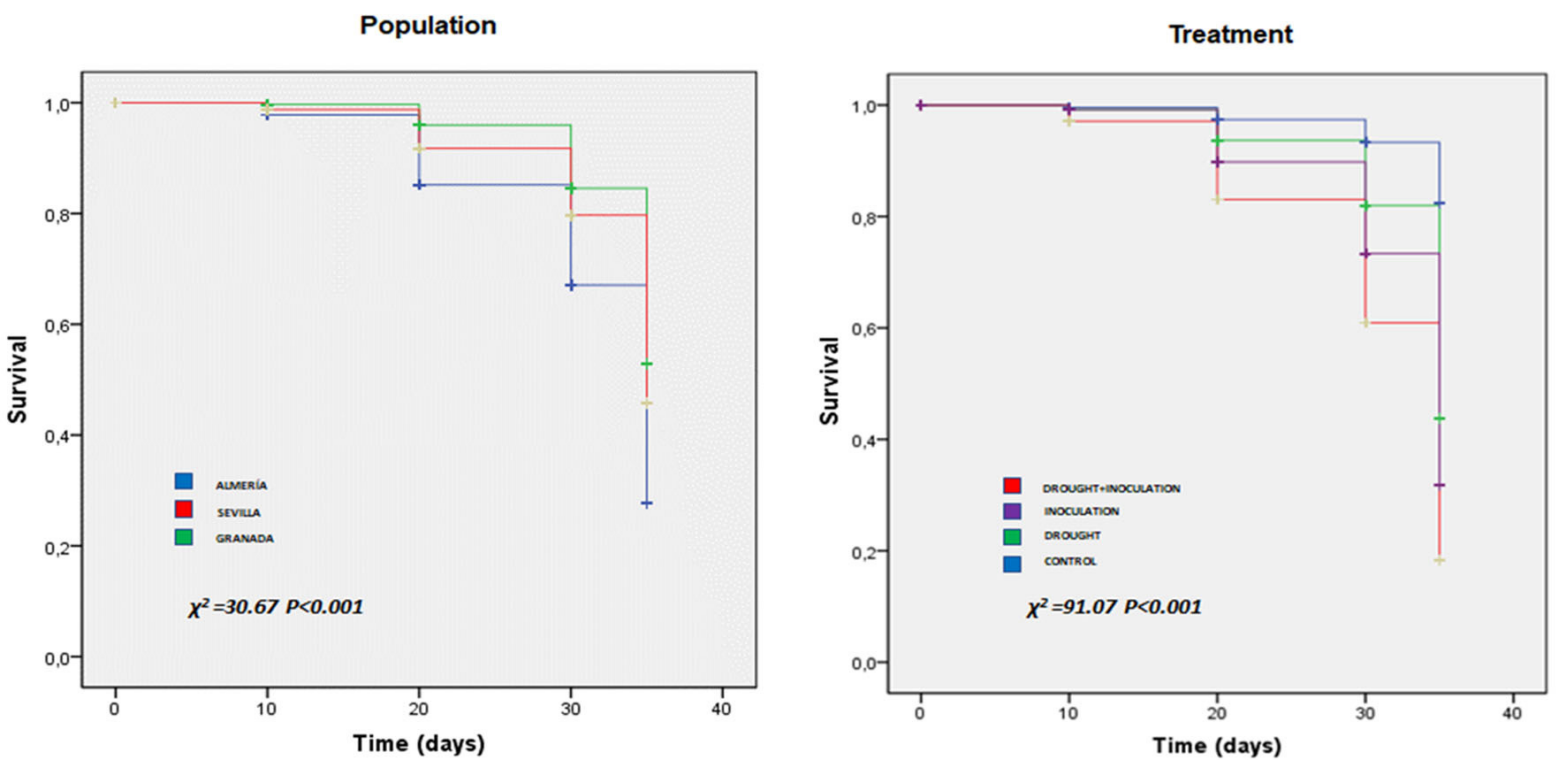
Survival plots for the three *Q. ilex* populations (Almeria, Granada, and Seville) under the individual and combined effects of inoculation with *P. cinnamomi* and drought as determined with the Kaplan–Meier model. The units of the *x*- and *y*-axis are days and seedling survival rate, respectively.

The effects of the two stresses were examined by inspecting leaf damage visually and recording the number of dead seedlings throughout the experiment. Leaf damage symptoms caused by both stresses included chlorosis, necrosis, and wilting, which were not observed in control seedlings (Figure 1). Damage appeared earlier and was more marked in the presence of both stresses. Thus, damage by the effect of combined stress appeared as early as day 6 in the Al population. Survival at the end of the experiment in the seedlings exposed to *P. cinnamomi* ranged from 50% in inoculated seedlings in the Se population to 0% in seedlings under double stress in the Al population. Based on Kaplan–Meier estimates, survival differed significantly among populations and also among treatments (*p* < 0.001 in both cases; Figure 2). The Al population had the smallest number of living seedlings, followed by the Se and Gr populations. Also, the combined treatment led to the lowest survival, followed by the inoculation, drought, and control treatment in this sequence.

### Physiological Parameters

Mean RLWC at the end of the experiment (day 35) ranged from 101.74% with the control treatment in the Se population to 44.22% with the combined treatment in the Al population (Supplementary Figure 4). However, no significant differences among populations (*p* = 0.3037) were observed. RLWC was significantly decreased (*p* < 0.001) by both the inoculation (65–70%) and combined treatments (44–76%), followed by the drought (75–85%) and control treatments (90–100%) (Supplementary Figure 4).

**Figure 3:**
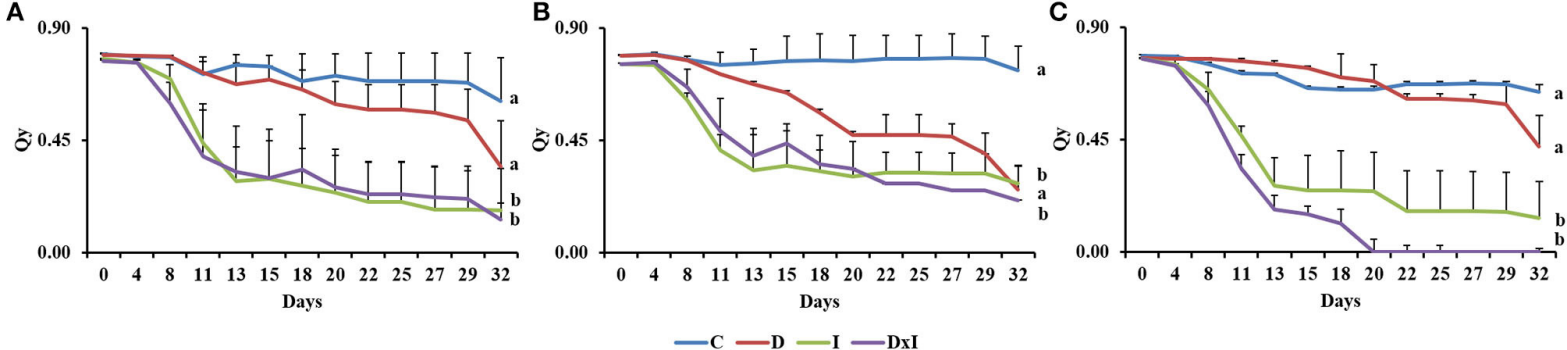
Quantum yield of photosystem II (Qy) in the dark of adapted leaves from the three *Q. ilex* populations (**A**, Seville; **B**, Granada; **C**, Almeria) under the control (C), drought (D), inoculation (I), and combined treatment (D×I). Values are mean ± SE for three biological replicates. Different letters, calculated from the area under the Qy curve, denote significant differences among treatments (*p* < 0.05).

The Qy in the control seedlings as determined in the dark remained nearly constant at 0.60–0.80 throughout the experiment (Figure 3). By contrast, Qy decreased gradually in the seedlings under the drought treatment. The pathogen, both by itself and in combination with drought, resulted in a marked decrease in Qy until day 13, after which the Qy leveled off at ~0.19. There were significant differences (*p* < 0.001) in this respect among treatments, the highest Qy levels corresponding to the inoculation and combined treatments. Also, although no significant differences among populations were observed (*p* = 0.5032), Al seedlings under the combined treatment behaved differently in this respect from Se and Gr seedlings, with near-zero Qy mean values by day 20.

**Figure 4:**
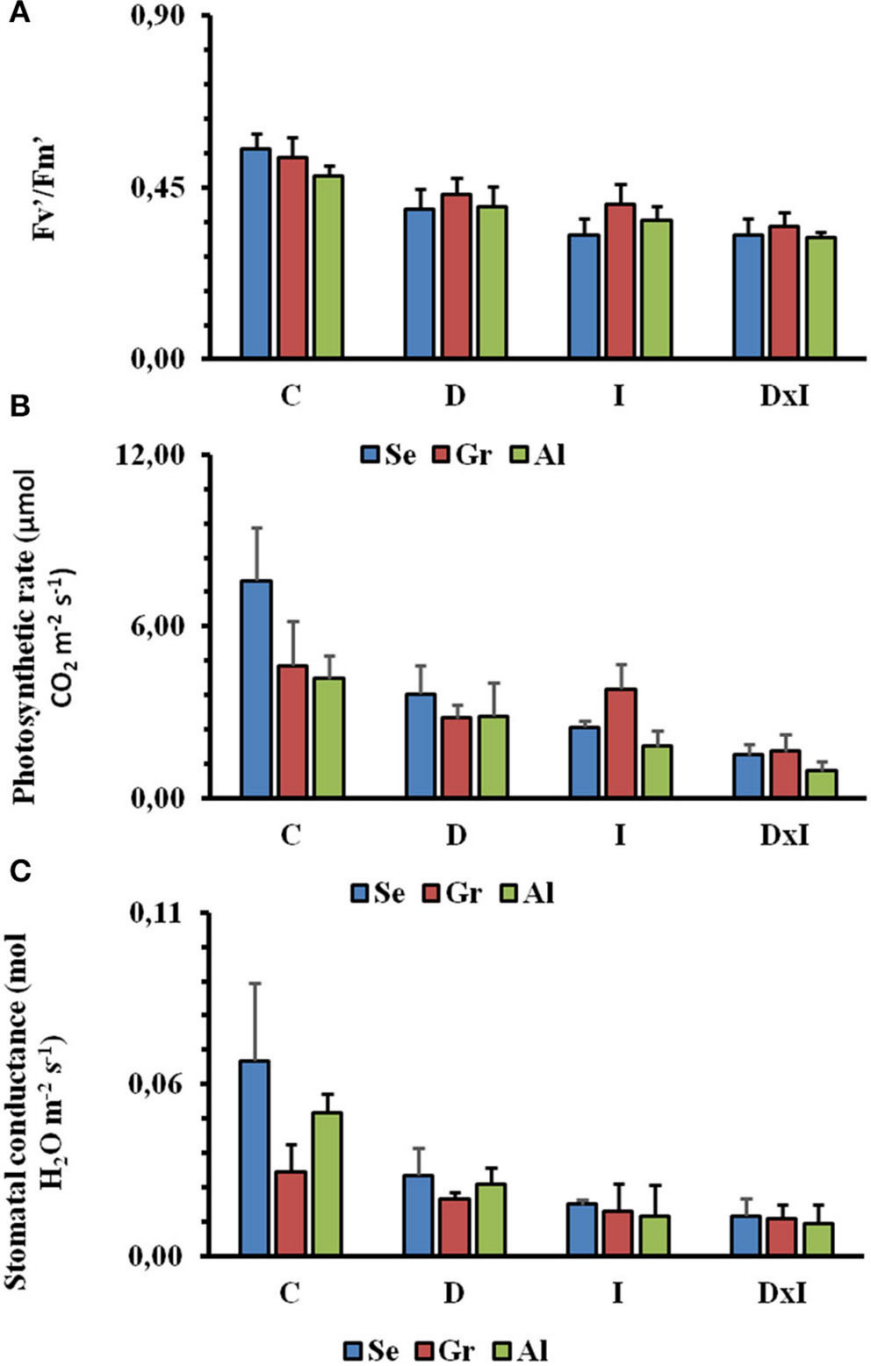
Mean values of $F_{v}^{\prime }$/$F_{m}^{\prime }$ **(A)**, net photosynthesis rate **(B)**, and stomatal conductance **(C)** in the Seville (Se), Granada (Gr), and Almeria (Al) populations from day 8 to day 32. Treatments: control (C), drought (D), inoculation (I), and combined (D×I). Different letters denote significant differences among treatments (*p* < 0.05).

Regarding photosynthetic activity determined on the IRGA analyzer, *Fv*′/$F_{\text{m}}^{\prime }$, a measure of photosynthetic efficiency in light-adapted leaves, was reduced by about 63% in the control seedlings from day 8 to day 32 (Figure 4A). There were, however, no statistically significant differences among populations or measurement times (*p* = 0.958). The decrease in $F_{\text{v}}^{\prime }$/$F_{\text{m}}^{\prime }$ was found to significantly depend on the particular treatment (*p* < 0.001), the combined effect being greater than those of the individual stresses. Reductions in the other photosynthetic parameters (A and Gs) were similar from day 8 to day 32 (Figures 4B,C). Thus, A was decreased from 5.47 to 1.37 μmol CO_2_ m^−2^ s^−1^ and Gs from 0.05 to 0.01 mol H_2_O m^−2^ s^−1^ over the period from day 8 to day 32. The low values of gas exchange in control seedlings were due to the low values of light intensity (900 μmol m^−2^ s ^−1^) through the experiment. Again, there were no statistically significant differences among populations or times. The two stresses, both individually and in combination, significantly decreased the previous parameters (*p* < 0.001). The effect, however, was especially marked with the inoculation of the pathogen.

### Changes in Photosynthetic Pigments and Metabolites

**Figure 5:**
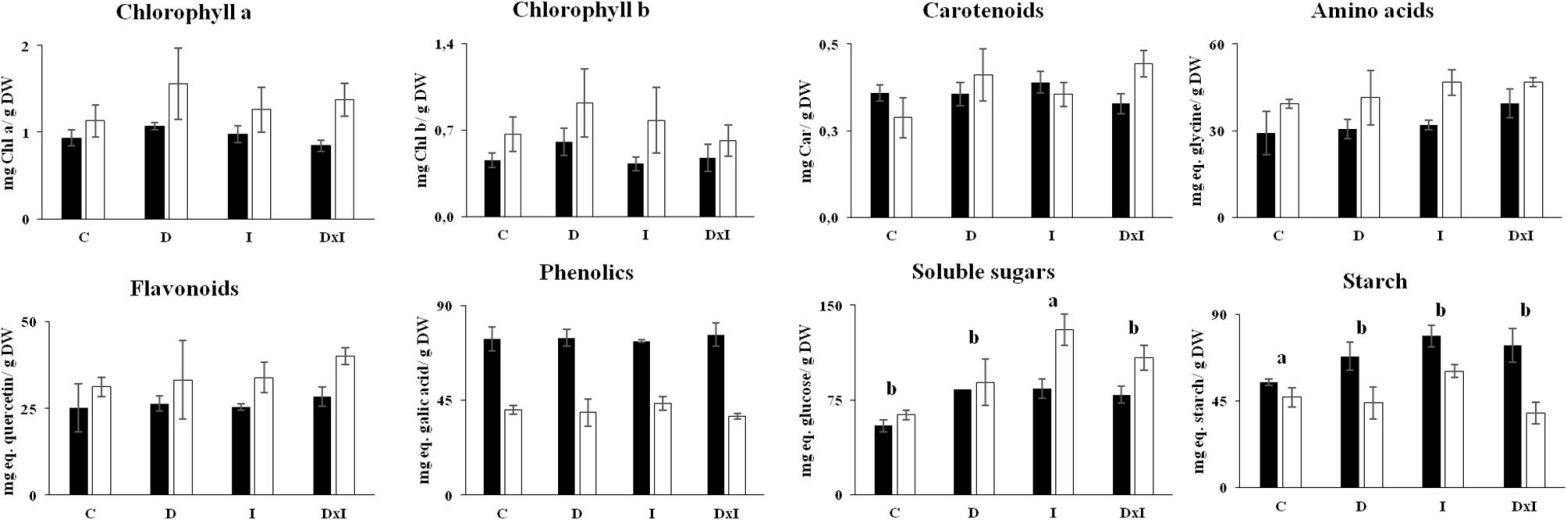
Contents in photosynthetic pigments (chlorophyll a, chlorophyll b, and carotenoids) and metabolites (amino acids, flavonoids, phenolics, soluble sugars, and starch) in leaves of *Q. ilex* seedlings from the Seville (white bars) and Almeria populations (black bars). Values are mean ± SE of three biological replicates on day 32. Different letters denote significant differences among treatments (*p* < 0.05).

The results of Figures 2, 3 led us to subject seedlings from the Se and Al populations, which were the most contrasting among the three, to biochemical and proteomic analyses. The determinations included photosynthetic pigments and metabolites (viz., amino acids, sugars, starch, phenolics, and flavonoids) in leaves on day 32. As can be seen from Figure 5, the contents in photosynthetic pigments of the control seedlings in the Se population exceeded those in the Al population. The respective contents in chlorophyll a, in milligrams per gram of dry weight, were 1.56 ± 0.41 and 0.84 ± 0.06; and those in chlorophyll b 0.92 ± 0.28 and 0.43 ± 0.05. There were no statistically significant differences in chlorophyll a (*p* = 0.5572) or chlorophyll b contents (*p* = 0.5431) among treatments. An identical trend was observed in amino acids, with higher contents in the Se population and no significant differences among treatments (*p* = 0.3451). Sugar contents were higher in the Al population than in the Se population; also, although all treatments increased them, the effect was more marked with inoculation alone than with drought alone or the combined treatment. Conversely, starch content was more abundant in the Al population, its highest contents corresponding to the drought, inoculation, and combined treatments. By contrast, none of the treatments changed the contents in phenolics and flavonoids.

### Proteomic Analysis

The total amount of proteins extracted with TCA/acetone–phenol and quantified with the Bradford method were similar (0.6–0.9 mg/g FW) in the Se and Al populations irrespective of treatment. 1-D SDS-PAGE electrophoresis resolved up to 37 bands that were present in seedlings from both populations, whichever the treatment (Supplementary Figure 5). Twenty-four bands differed statistically among treatments, nine differing within each population and six in both. Most of the resolved bands (11 in total) were more abundant with the combined treatment in at least one population (Supplementary Table 1). Based on the PCA results, PC1, which explained 40.5% of the variability in the Se population, discriminated the control and drought treatments on the one hand, and the inoculation and combined treatment on the other. In the Al population, however, PC1, which explained 37.5% of variability, only discriminated the combined treatment (Supplementary Figure 5).

Proteins differing between populations and/or treatments were identified by using shotgun analysis, a powerful proteomics platform. The results are summarized in Table 2. Filtering the original dataset (3,412 and 2,600 positive matches in the Se and Al population, respectively) for confident matches (≥ 2 peptides, ≥ 2 score, and ≥ 15% coverage, only those proteins consistently present in the three biological replicates, standard deviation <50%) provided 414 confidence proteins in the Se population and 734 in the Al population, 318 being shared by the two. Statistical analysis for variable proteins reduced the original dataset to 83 in Se and 223 in Al, 25 being shared by the two populations. The whole dataset of confident proteins was categorized in functional terms by using MERCATOR, which established 17 groups (Supplementary Figures 6, 7). The best represented groups were carbohydrate metabolism (78 proteins in Se and 116 in Al); folding, sorting, and degradation (75 and 141, respectively); energy metabolism (31 and 67); amino acid metabolism (37 and 53); secondary metabolism (29 and 45); ROS scavenging (24 and 18); cellular processes (19 and 31); and defense (12 and 27) (Supplementary Figure 6).

**Table 2:** Summary of proteins identified by the shotgun–MS/MS analysis in the two populations and their combination.

	**Seville**	**Almeria**	**Both**
Raw data	3,412	2,600	2,484
Confidence parameters (≥ 2 Peptides, Score ≥ 2, Coverage ≥ 15%)	2,447	2,214	2,175
Consistent proteins	1,110	1,214	1,015
Standard deviation <50% between replicates	414	734	318
Statistically significant (*p* ≤ 0.05)	83	223	25

**Figure 6:**
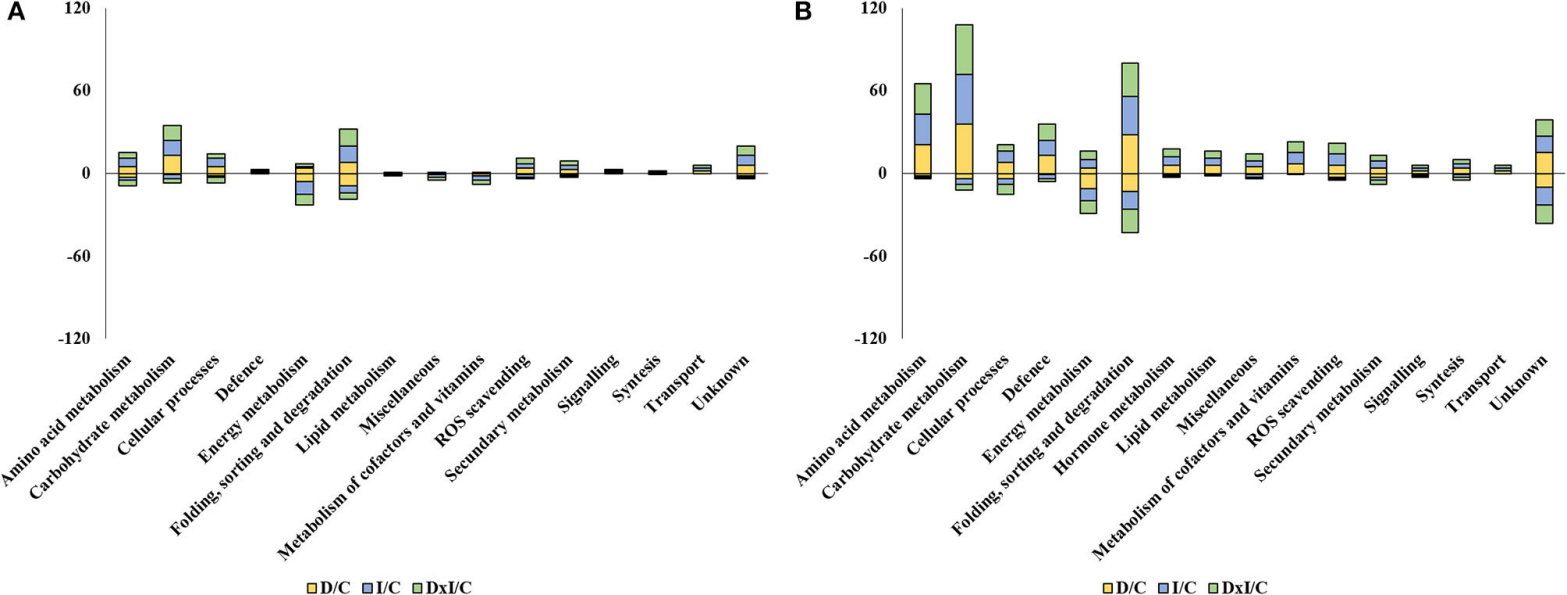
Number of up- and down-accumulated variable proteins whose abundance in the drought (D), inoculation (I), and combined (D×I) treatments was greater or less than with the control treatment (C) in the Seville **(A)** and Almeria **(B)** populations. The numbers of up- and down-accumulated proteins are shown as positive and negative values, respectively.

Variable proteins fell in 15 groups in Se and 16 in Al, the best represented groups being folding, sorting, and degradation (17 and 41, respectively); carbohydrate metabolism (14 and 40); energy metabolism (10 and 15); amino acid metabolism (8 and 23); ROS scavenging (5 and 9); and secondary metabolism (4 and 7) (Supplementary Figure 6). All functional groups present in the dataset of variable proteins included more upaccumulated proteins—by exception, the energy metabolism group comprised more downaccumulated proteins in both populations (Figure 6). Based on the number of variable proteins, the proteome was more markedly affected by the treatments in Al than it was in Se (173 vs. 45 specific proteins). No treatment-related qualitative differences were observed; however, in any case, the combined treatment induced more proteome changes than did the individual stresses in the Se population; also, the presence of *P. cinnamomi*, both individually and in combination with drought, led to more marked changes in the Al population (Supplementary Table 1).

**Figure 7:**
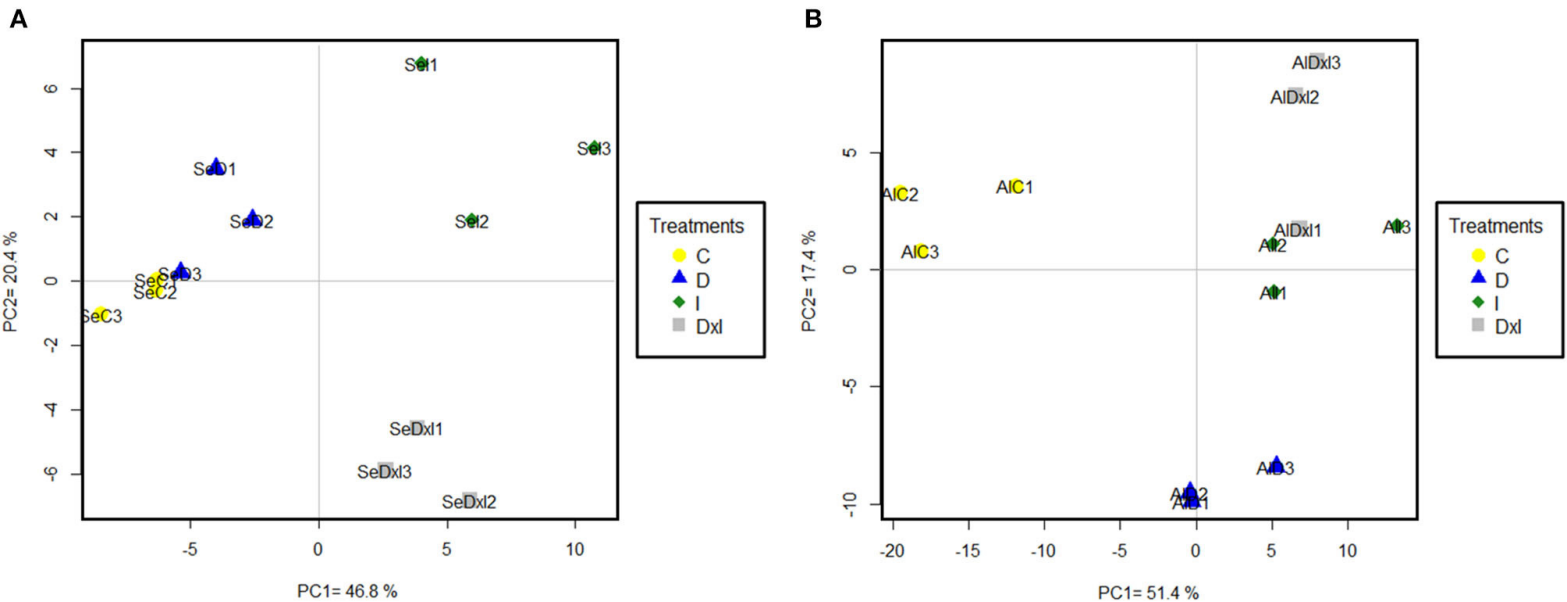
Principal component analysis of variable proteins identified in the Seville **(A)** and Almeria **(B)** populations. Treatments: C control; D drought; I inoculation; D×I combined. Numbers indicate biological replicates for each treatment.

The previous datasets were simplified by PCA. Using the whole set of confidence proteins did not allow separation among populations or treatments with either PC1 or PC2, which accounted for 26.00% and 15.20% of the variability in the data (Supplementary Figure 8). Differences among treatments in each population were exposed by subjecting the dataset of variable proteins to PCA (Figure 7). In Se, PC1 (46.80% variability) discriminated the control and drought treatments from the inoculation and combined treatments, while PC2 (20.40% variability) discriminated the latter two. In Al, PC1 (51.40%) discriminated the control treatment from all others and PC2 (17.40%) discriminated drought from the pathogen. The variable proteins most markedly contributing to PC1 and PC2 are listed by the functional group in Table 3 and Supplementary Table 2. In both populations, the greatest positive loadings were those of the carbohydrate metabolism, and folding, sorting, and degradation groups. On the other hand, the majority of the proteins related to energy metabolism that most markedly contributed to both PC were involved in photosynthesis, such as photosystem II subunit P-1, ferredoxin–NADP reductase, postillumination chlorophyll florescence increase protein, 33 kDa oxygen-evolving protein of photosystem II, phosphoglycolate phosphatase 1A, PsbP domain-containing protein 3, and sedoheptulose-1,7-biphosphatase (Supplementary Table 2).

**Table 3:** First 20 proteins with positive loadings contributing to PC1 and PC2 in the Seville (Se) and Almeria (Al) populations as classified by functional group.

**Functional group**	**Protein**	**Up-accumulated**	**Loadings**
**Se POPULATION**			
**PC1**			
Amino acid metabolism	Cysteine synthase	I	0.134671201
	Alanine–glyoxylate aminotransferase 2 homolog 1, mitochondrial	I; D×I	0.138725675
Carbohydrate metabolism	6-phosphogluconate dehydrogenase, decarboxylating	I; D×I	0.138255713
	Citrate synthase	I	0.146813054
	Aldehyde dehydrogenase, mitochondrial	I; D×I	0.150395963
Cellular processes	Putative reversibly glycosylatable polypeptide	I	0.145130599
	α-1,4-glucan-protein synthase [UDP-forming] 2	I; D×I	0.148753876
Folding, sorting, and degradation	Proline iminopeptidase	I	0.128417337
	Eukaryotic translation initiation factor 3 subunit F	I	0.133480073
	ATP-dependent Clp protease proteolytic subunit-related protein 1, chloroplastic	I	0.138556079
	40S ribosomal protein S5	I	0.139542434
	Translocase of chloroplast	D×I	0.145884352
ROS scavenging	FrnE protein-like	I; C	0.133528064
Secondary metabolism	NADPH-dependent codeinone reductase	D×I	0.141369245
	NADPH:protochlorophyllide oxidoreductase porA	I; D×I	0.142549926
Signaling	Trans-2-enoyl-CoA reductase, mitochondrial	I; D×I	0.130723221
Transport	V-type proton ATPase subunit C	I	0.143391600
Unknown	Outer envelope pore protein 37, chloroplastic	I	0,134589518
	BnaA01g04430D protein	I; D×I	0,138201036
	Carboxylate clamp-tetratricopeptide repeat protein	I; D×I	0,138579354
**PC2**			
Amino acid metabolism	Glyoxalase I	C; D; I	0.203639693
	Arginine biosynthesis bifunctional protein ArgJ	I	0.204108147
Carbohydrate metabolism	Fructokinase	I	0.120840694
	α-amylase	I	0.134587804
	HMG aldolase	C; D; I	0.191073158
Cellular processes	Cell division protein FtsZ	C	0.121972298
	Patatin	I	0.130841894
	Cell division protein FtsZ homolog 1, chloroplastic	C; D; I	0.153978976
	Harpin binding protein 1	C; D; I	0.215407463
Energy metabolism	Photosystem II subunit P-1	C	0.114099595
Folding, sorting, and degradation	Translocase of chloroplast	I	0.136243712
	Mitochondrial processing peptidase	I	0.163693915
	Mitochondrial processing peptidase subunit beta	I	0.170347898
	Peptidyl-prolyl *cis-trans* isomerase	C; D; I	0.173743165
	Clone PI4869 proteasome inhibitor-like protein mRNA	I	0.214512684
Miscellaneous	Oxidoreductase, putative	I	0.200450416
Secondary metabolism	Caffeic acid *O*-methyltransferase	I	0.121922782
Unknown	Cysteine synthase	I	0,119390920
	Fructose-bisphosphate aldolase	I	0,129593872
	Lysine 6-aminotransferase	I	0,173751918
**Al POPULATION**			
**PC1**			
Carbohydrate metabolism	Glucose-6-phosphate 1-dehydrogenase, cytoplasmic isoform 2	D×I	0.083620512
	Aldehyde dehydrogenase	D×I; I; D	0.083884994
	Serine hydroxymethyltransferase	D×I	0.083941959
	Malic enzyme	D×I; I	0.084150059
	NADH dehydrogenase [ubiquinone] flavoprotein 2, mitochondrial	D×I; I; D	0.084854012
	ATP synthase subunit alpha, mitochondrial	D×I; I; D	0.085624630
	Formate dehydrogenase	D×I; I; D	0.085695392
	Malic enzyme	D×I; I	0.086636987
	Glucose-1-phosphate adenylyltransferase large subunit, chloroplastic/amyloplastic	I	0.086639036
	Malate dehydrogenase	D×I; I; D	0.088034545
Cellular processes	UDP-D-apiose/UDP-D-xylose synthase	I	0.084723943
	α-1,4-glucan-protein synthase [UDP-forming] 2	D×I; I; D	0.086561347
Energy metabolism	Fructose-bisphosphate aldolase	D×I; I; D	0.086927615
Folding, sorting, and degradation	Heat shock protein 60	D×I; I; D	0.085004175
	T-complex protein 1 subunit zeta 1	D×I; I; D	0.089137549
Hormone metabolism	Aluminum-induced protein with YGL and LRDR motifs	D×I; I; D	0.084157903
	Probable aldo–keto reductase 1	D×I; I; D	0.085397904
Lipid metabolism	Acyl-[acyl-carrier-protein] desaturase	I	0.087659507
Miscellaneous	Dynamin-related protein 1E	D×I; I	0.086364345
ROS scavenging	Monothiol glutaredoxin-S17	D×I; I; D	0.083444644
**PC2**			
Amino acid metabolism	Ornithine carbamoyltransferase, chloroplastic	I; D×I	0.067037801
	Methionine synthase	D×I	0.086406313
Carbohydrate metabolism	Aldehyde dehydrogenase, mitochondrial	D×I	0.069051721
	Glucose-6-phosphate isomerase 1, chloroplastic	D×I	0.089556889
	Serine hydroxymethyltransferase	D×I	0.094407201
	Carbonate dehydratase	D×I	0.105989354
Defense	BURP domain protein RD22	D×I	0.086388976
Energy metabolism	Ferredoxin–NADP reductase	D×I	0.102371216
	Serine–glyoxylate aminotransferase	D×I	0.123546721
Folding, sorting, and degradation	40S ribosomal protein S19	D×I	0.068660324
	AMPP	D×I	0.075187168
	50S ribosomal protein L5, chloroplastic	D×I	0.087674819
	60S ribosomal protein L17-2	D×I	0.104318614
Hormone metabolism	12-oxophytodienoate reductase 1	I; D×I	0.068398959
Miscellaneous	CTF2A-like oxidoreductase	D×I	0.098176129
Nucleotide metabolism	Adenylate kinase	I; D×I	0.102221691
ROS scavenging	Catalase	I; D×I	0.109176303
	Catalase	I; D×I	0.118027832
Secondary metabolism	Aminotransferase	I; D×I	0.073624373
Signaling	Sulphite reductase 1 [ferredoxin], chloroplastic	D×I	0.099056469

*C, D, I, and D×I indicate control, drought, inoculation, and combined treatment, respectively*.

## Discussion

In nature, plants are simultaneously exposed to a combination of biotic and abiotic stresses (Ramegowda and Senthil-Kumar, 2015; Pandey et al., 2017; Teshome et al., 2020). In a previous study, our group used molecular methods to investigate the individual effects of *P. cinnamomi* and drought, which are the two main stresses leading to oak decline (Jorge et al., 2006; Echevarría-Zomeño et al., 2009; Sghaier-Hammami et al., 2013; Valero-Galván et al., 2013; Simova-Stoilova et al., 2015, 2018; San-Eufrasio et al., 2020), on *Q. ilex* seedlings and their response to these two stresses. In this work, we went one step forward by exploring the combined effects of abiotic (water withholding) and biotic stress (*P. cinnamomi* inoculation) on *Q. ilex* seedlings from three contrasting Andalusian populations (Se, Gr, and Al). Only one study of the effects of combined stress on seedling traits and physiology in holm oak had previously been reported which, however, failed to examine metabolic changes or consider the potential influence of plant variability (Ruiz-Gómez et al., 2018). The high interpopulation and intrapopulation variability of *Q. ilex* (Valero-Galván et al., 2011; San-Eufrasio et al., 2020) led us to compare several Andalusian populations of this species to gain further insight into the response of holm oak to *P. cinnamomi* and drought.

The effects of stresses and the ensuing response are known to depend on stress intensity and duration. In this work, infection of seedlings was confirmed by isolating the pathogen and identifying it under a light microscope at the end of an experiment where seedlings were placed under severe drought conditions (see San-Eufrasio et al., 2020).

### Plant Mortality and Physiological Response

The effect and the level of tolerance to the stresses were assessed here through the visual inspection of the damage symptoms and the number of dead seedlings, which differed among populations and treatments. As regards variability in resilience among populations or individuals within populations, the combined effects of *P. cinnamomi* attack and drought are more damaging to survival than are those of the two stresses in isolation (Desprez-Loustau et al., 2006; Sherwood et al., 2015; Ghanbary et al., 2017, 2020; Ruiz-Gómez et al., 2018). Response to an attack by *P. cinnamomi* drought stress was first observed in the Al population, which is located in the eastern part of Andalusia. This population is in the farthest region to the place where *P. cinnamomi* root rot was first observed, which was seemingly the southwest of the Iberian Peninsula (Brasier, 1996). This is consistent with the increased susceptibility to *P. cinnamomi* of eastern Andalusian populations previously reported by Sghaier-Hammami et al. (2013). On the other hand, *Q. ilex* populations located in the eastern part were previously found to be more tolerant of drought than those in the western part (Valero-Galván et al., 2013; Navarro-Cerrillo et al., 2018), the combination of both stresses having a greater impact on Al than on the other two populations studied here. In turn, the Se and Gr populations exhibited a similar response to *P. cinnamomi* and drought in terms of damage symptoms and mortality, the earliest symptoms of seedling damage being observed on day 6 in both populations. The Gr population responded more effectively to the individual and combined effects of the two factors; in fact, it was the Gr population that exhibited the highest seedling survival at the end of the experiment.

The effects of both stresses and the response of *Q. ilex* to them were also assessed in physiological terms through water status and photosynthetic activity, as previously carried out by Sghaier-Hammami et al. (2013) and Valero-Galván et al. (2013) in studying individual sources of stress. RLWC was significantly decreased by drought and *P. cinnamomi*, both individually and in combination, relative to the control seedlings, the effect of the pathogen attack being especially marked (De Pascali et al., 2019). However, undamaged *Q. ilex* seedlings maintained their leaf moisture levels, which suggest that they succeeded in holding leaf turgor after 35 days under stressing conditions (Forner et al., 2018). The maximum PSII photochemical efficiency (Qy) and conversion efficiency of PSII open reaction centers ($F_{\text{v}}^{\prime }$/$F_{\text{m}}^{\prime }$) are measures of photochemical activity, commonly used as parameters of response to stress in plants (Bolhàr-Nordenkampf and Öquist, 1993; Filella et al., 1998; Peguero-Pina et al., 2009; Murchie and Lawson, 2013; Sancho-Knapik et al., 2018; Jia et al., 2019). Photosynthetic activity decreased throughout the experiment but differences among populations were not significant (San-Eufrasio et al., 2020). This indicates that the PSII reaction site in *Q. ilex* leaves was affected by the two stresses, which inhibited photosynthesis in the seedlings. Also, the fact the combined treatment led to the lowest chlorophyll fluorescence values at the end of the experiment in the Al population is suggestive of an especially synergistic effect of the two stresses on photosynthesis in this population.

An early, fast decrease in A) and Gs) was observed in seedlings under the individual and combined action of *P. cinnamomi* and drought in all populations, which exhibited low values of the two parameters throughout the experiment (Maurel et al., 2001; Sghaier-Hammami et al., 2013; Corcobado et al., 2014; Merilo et al., 2014; Ruiz-Gómez et al., 2018). Early quick stomatal closure in response to *P. cinnamomi* and drought may have reduced water losses and carbon dioxide uptake (Lawlor and Cornic, 2002; Merilo et al., 2014). The quantitative response of these physiological parameters to the individual stresses and their combination was population-independent; in any case, there was a stronger, but not statistically different, response to combined stress (Ruiz-Gómez et al., 2018). Unlike previously found by Ruiz-Gómez et al. (2018), infection by *P. cinnamomi* had more marked effects than drought, possibly as a consequence of the pathogen damaging the root system and reducing water uptake as a result (Crombie and Tippett, 1990; Corcobado et al., 2013). This reduction in turn may have increased water deficiency, thereby decreasing physiological parameters in the seedlings (Maurel et al., 2001; Robin and Desprez-Loustau, 2001). Overall, the physiological response of *Q. ilex* to attack by *P. cinnamomi* and drought, whether individually or in combination, was quite similar among populations, the photosynthetic apparatus of the seedlings being affected mainly by the combination of the two stresses.

### Biochemical Parameters in Undamaged Seedlings

The fact that the contents in photosynthetic pigments of undamaged seedlings under stress from drought and *P. cinnamomi* inoculation were similar to those of the control seedlings suggests that the photosynthetic apparatus was altered by neither source of stress (Gallé et al., 2007; San-Eufrasio et al., 2020). Thus, neither amino acids nor phenolics nor flavonoids among other biomolecules were altered in their contents under the stress conditions. The contents in soluble sugars and starch increased markedly in seedlings under both sources of stress, followed by those under drought alone. Abiotic and biotic stresses are known to increase the soluble sugar contents of leaves by regulating expression in genes involved in photosynthesis, osmolyte synthesis, and sucrose metabolism (Holland et al., 2016; Khan et al., 2020). Beyond its role as a source of carbon and energy *via* fermentative or aerobic pathways, soluble sugars promote water uptake to maintain cell volume while avoiding wilting (Manes et al., 2006; Holland et al., 2016). In this work, we found increased starch accumulation with all treatments relative to the control seedlings, the starch content being especially high in Al seedlings under the inoculation or combined treatment. Starch, phenolic compounds, and other defense-related substances were previously found to accumulate in xylem and protoxylem cells in the roots of inoculated plants (Ruiz Gómez et al., 2014; Redondo et al., 2015). This is consistent with the results of Sghaier-Hammami et al. (2013), who found an increased abundance of proteins involved in starch biosynthesis in response to an attack by *P. cinnamomi*. Therefore, although our seedlings responded more strongly to the pathogen than to drought, their metabolism was not imbalanced as a result. In conclusion, the seedlings succeeded in maintaining cellular homeostasis beyond physiological disturbances even under severe stress conditions.

### Alteration of the Protein Profile by Drought and *P. cinnamomi*

The proteomic techniques used (1D and shotgun-MS/MS analysis) allowed the identification and quantification of a large set of proteins in *Q. ilex* seedling leaves altered by inoculation with *P. cinnamomi* and/or drought. Based on the number of 1D SDS-PAGE bands and their intensity, the combined treatment had a stronger effect than all others; also, the Al population was more markedly affected than the Se population, which is quite consistent with the previous results. Based on the shotgun results, the Al population exhibited more changes in both confidence and variable proteins than did the Se population. This was a consequence of the above-described results as regards damage symptoms and mortality and suggests that the most susceptible population was that undergoing the greatest proteomic changes. The differences in the abundance of proteomic changes were found to be dependent on the particular population. Thus, the two populations differed in the number of proteins but not in the functional categories. The largest groups of confidence and variable upaccumulated proteins in the two populations were those of amino acid metabolism; carbohydrate metabolism; folding, sorting, and degradation; ROS scavenging; and secondary metabolism (Sghaier-Hammami et al., 2013; Valero-Galván et al., 2013; Hildebrandt, 2018; San-Eufrasio et al., 2021). In contrast, the largest number of downaccumulated proteins in both populations, which contributed more to the variability, was that of the photosynthesis (Kapoor et al., 2020), which is in line with the above-described results for physiological parameters. Multivariate analysis revealed a different response to *P. cinnamomi* and drought in the two populations. PCA allowed effective discrimination of the inoculation and combined treatments from the drought and control treatments in the Se population, and also of the control treatment from the individual and combined treatments in the Al population. In addition, PCA clearly discriminated between drought and *P. cinnamomi*, the latter individually or in combination. Therefore, proteins in both populations were more markedly affected by *P. cinnamomi* attack than they were by drought.

### Putative Markers of Resilience to Stress

Those proteins that were consistently found in the three biological replicates from both populations contributed markedly to variability in the PCA and were more abundant in the combined treatment were taken to be putative molecular markers. The specific proteins considered were aldehyde dehydrogenase, glucose-6-phosphate isomerase, 50S ribosomal protein L5, and α-1,4-glucan-protein synthase [UDP-forming]. Aldehyde dehydrogenase levels are known to be raised by both abiotic and biotic stresses (Tola et al., 2021). Thus, plants under stress produce increased amounts of ROS that in turn boost aldehyde production by cells through stress-induced lipid peroxidation (Bartels and Sunkar, 2005; Tola et al., 2021). Aldehyde dehydrogenase detoxifies aldehydes by oxidizing them to carboxylic acids (Tola et al., 2021). Sunkar et al. (2003) found overexpression of aldehyde dehydrogenase under Arabidopsis conditions to increase tolerance of dehydration. The 50S ribosomal protein L5 is a chloroplast ribosomal protein that is upregulated in response to abiotic stress by boosting the synthesis of chloroplast-encoded proteins to offset damage in photosynthesis proteins caused by abiotic stress (Zhu et al., 2021). α-1,4-Glucan-protein-synthase, which is involved in the biogenesis or degradation of cell walls, has been identified in response to drought conditions (Fadoul et al., 2018; Dugasa et al., 2021). UDP-forming protein is associated with the formation of cell walls as physical barriers against pathogens (Shoresh and Harman, 2008). Glucose-6-phosphate isomerase (also called “phosphoglucose isomerase”), a glycolytic enzyme that interconverts glucose-6-phosphate and fructose-6-phosphate, is a drought stress-related protein whose synthesis is boosted under water-deficient conditions (Khanna et al., 2014). This enzyme has been deemed a promoter of starch synthesis by leaves (Backhausen et al., 1997; Yu et al., 2000). Therefore, all the proteins proposed as markers of resilience to combined biotic and abiotic stress, and hence to the decline syndrome in *Q. ilex* are responsive to adverse environmental conditions, their increased production being a part of the survival mechanisms of this species under restrictive conditions.

## Conclusions

The presence of stress from *P. cinnamomi* and drought was found to have a synergistic effect on *Q. ilex* seedlings from three contrasting populations in Andalusia, southern Spain. There were no qualitative, but only quantitative differences in the effects and responses to the individual or combined stresses, the Al population being the most markedly affected by the combined treatment, and hence the theoretically most vulnerable to the decline syndrome. As regards individual stresses, drought had a more marked effect than *P. cinnamomi* on the Se and Gr populations, whereas the opposite held for the Al population. Even so, a variable proportion of seedlings from each population responded effectively to stress, with no visible symptoms of leaf damage at any time during the experiment. Those asymptomatic seedlings responded differently to the individual stresses and their combination. Thus, despite the reduced moisture content and photosynthetic activity, their levels were still high enough for cellular homeostasis to be maintained and differences in the contents of key biomolecules such as photosynthetic pigments, amino acids, and phenolics to be insubstantial. The protein functional groups undergoing the greatest changes were folding, sorting, and degradation; carbohydrate metabolism; amino acid metabolism; ROS scavenging and secondary metabolism (upaccumulated); and energy metabolism (downaccumulated). The reduction in photosynthetic activity may have arisen from an increase in heterotrophic catabolism. Also, stress-related proteins were more abundant in the Al population than they were in the other two. The following proteins are proposed as putative markers of resilience to the decline syndrome in *Q. ilex*: aldehyde dehydrogenase, glucose-6-phosphate isomerase, 50S ribosomal protein L5, and α-1,4-glucan-protein synthase [UDP-forming].

## Data Availability Statement

The original contributions presented in the study are publicly available. This data can be found at: MS proteomics raw data were deposited with dataset identifier PXD025704 in the ProteomeXchange Consortium via the PRIDE partner repository (Perez-Riverol et al., 2019).

## Author Contributions

JJ-N and M-DR conceived and designed the experiments. BS-E and M-DR performed the experiments. FR-G supplied the *P. cinnamomi* strain, provided the acorns, and helped with the greenhouse measurements. BS-E, MC, JJ-N, RN-C, and M-DR analyzed the data. JJ-N and M-DR wrote the manuscript. All authors read and approved the final manuscript.

## Conflict of Interest

The authors declare that the research was conducted in the absence of any commercial or financial relationships that could be construed as a potential conflict of interest.

## Publisher's Note

All claims expressed in this article are solely those of the authors and do not necessarily represent those of their affiliated organizations, or those of the publisher, the editors and the reviewers. Any product that may be evaluated in this article, or claim that may be made by its manufacturer, is not guaranteed or endorsed by the publisher.
