# Supplementary material for: Effect and Response of Quercus ilex subsp. ballota [Desf.] Samp. Seedlings From Three Contrasting Andalusian Populations to Individual and Combined Phytophthora cinnamomi and Drought Stresses
Source: Front Plant Sci. 2021 Aug 19;12:722802. doi: 10.3389/fpls.2021.722802 (PMC8417417; doi:10.3389/fpls.2021.722802)
Supplement: Supplementary file 1 [file Data_Sheet_1.docx]

**Effect and response of *Quercus ilex* subsp. *ballota* [Desf.] Samp. seedlings from three contrasting Andalusian populations to individual and combined *Phytophthora cinnamomi* and drought stresses**

Bonoso San-Eufrasio^1^, María Ángeles Castillejo^1^, Mónica Labella-Ortega^1^, Francisco Ruiz-Gómez^2^, Rafael M Navarro-Cerrillo^2^, Jesús V. Jorrín-Novo^1^, María-Dolores Rey^1^*

^1^Agroforestry and Plant Biochemistry, Proteomics and Systems Biology, Department of Biochemistry and Molecular Biology, University of Cordoba, UCO-CeiA3, 14014 Cordoba, Spain

^2^ Laboratory of Dendrochronology, Silviculture and Global Change, DendrodatLab-ERSAF Department of Forest Engineering, University of Cordoba, Campus de Rabanales, Ctra. N. IV, 14071 Cordoba, Spain

Supplementary Material

**
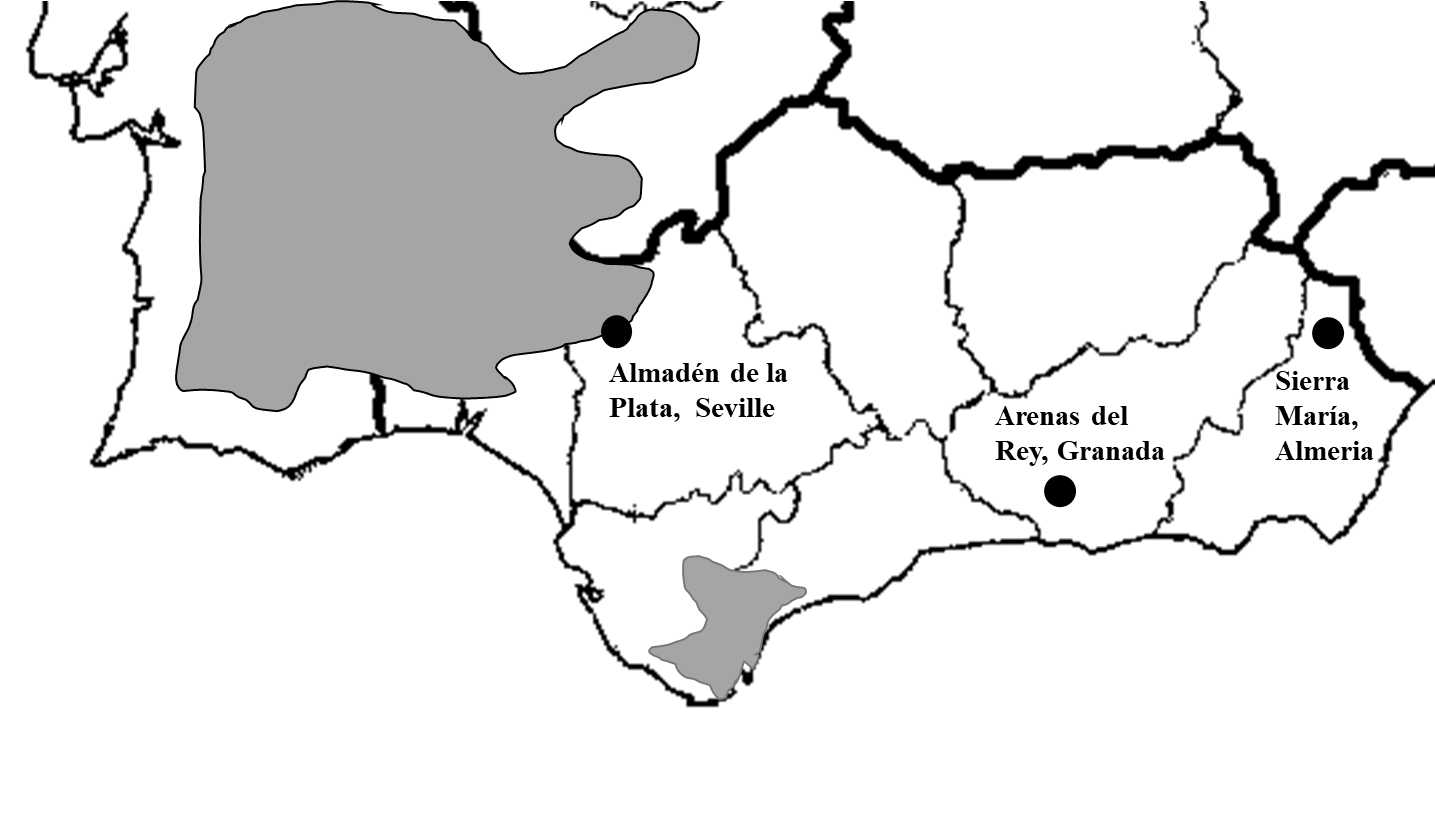
**

**Supplementary Figure 1| Localization of the three Andalusian populations used in this work and distribution of decline sites of *Q. ilex* (presence of *P. cinnamomi*) located in Portugal and Spain.** Adapted from Brasier, 1993.


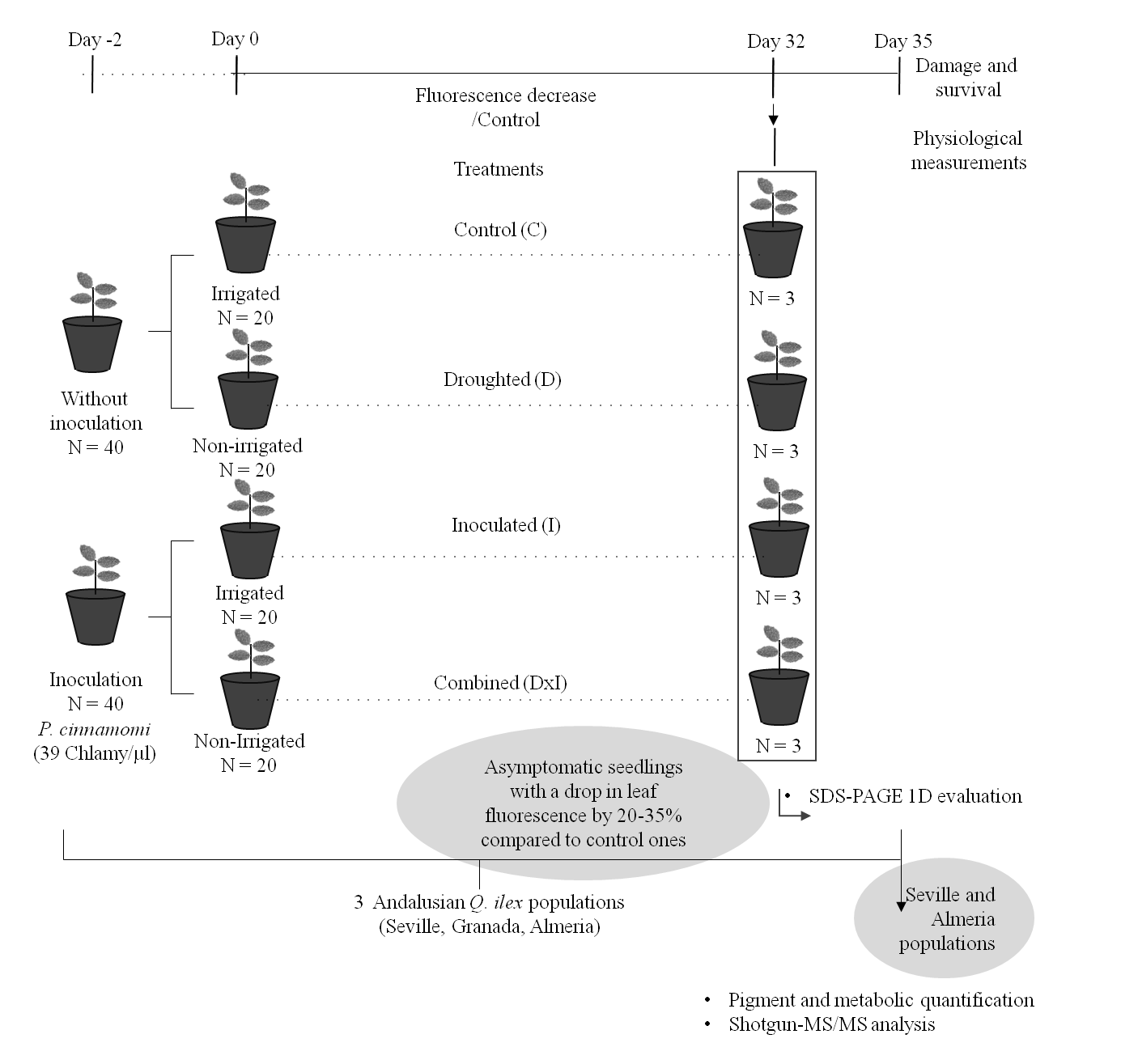


**Supplementary Figure 2|** **Experimental design used to examine the individual and combined effects of drought and *P. cinnamomi* on *Q. ilex* seedlings.** N number of biological replicates. Treatments: C control; D drought; I inoculation; D**×**I combined


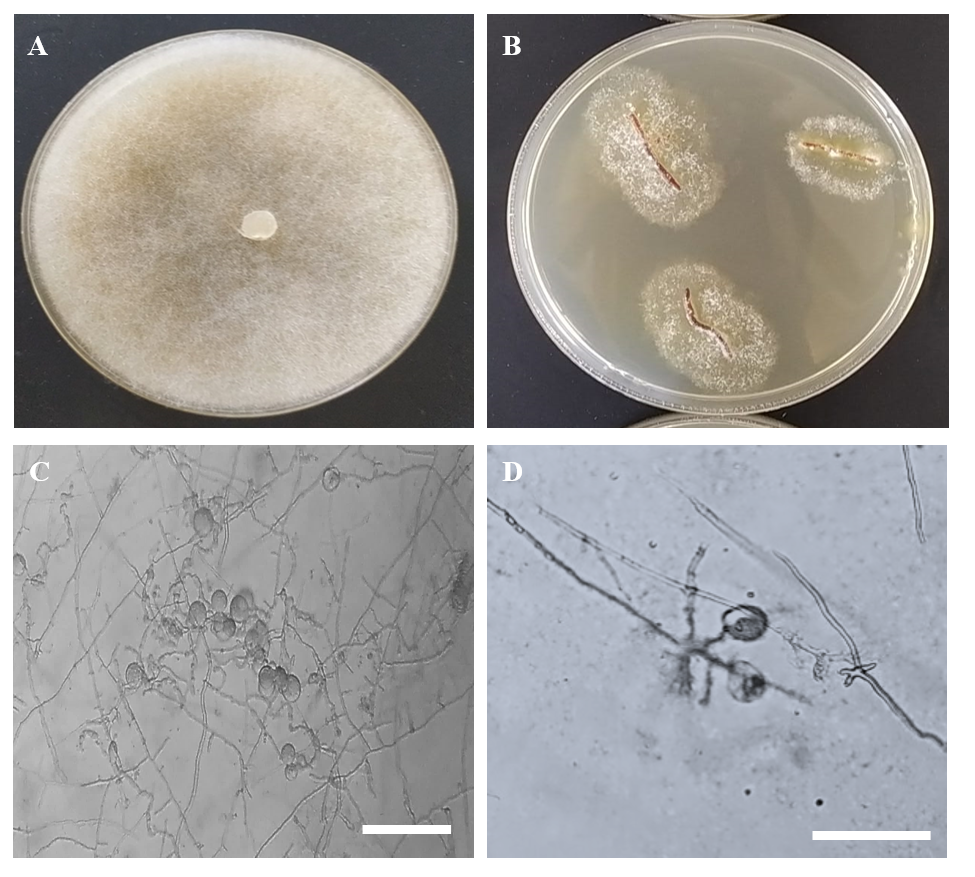


**Supplementary Figure 3| Morphological structures of *Phytophthora cinnamomi*.** (**A**) Seven days old colonies on carrot–agar medium. (**B**) *P. cinnamomi* isolation from root cuts of Holm oak seedling inoculated with the pathogen on PARPBH selective medium. (**C**) Microscopic view of *P. cinnamomi* mycelium with chlamydospores. Scale bar: 10 µm. (**D**) Mature zoosporangium. Scale bar: 20 µm.


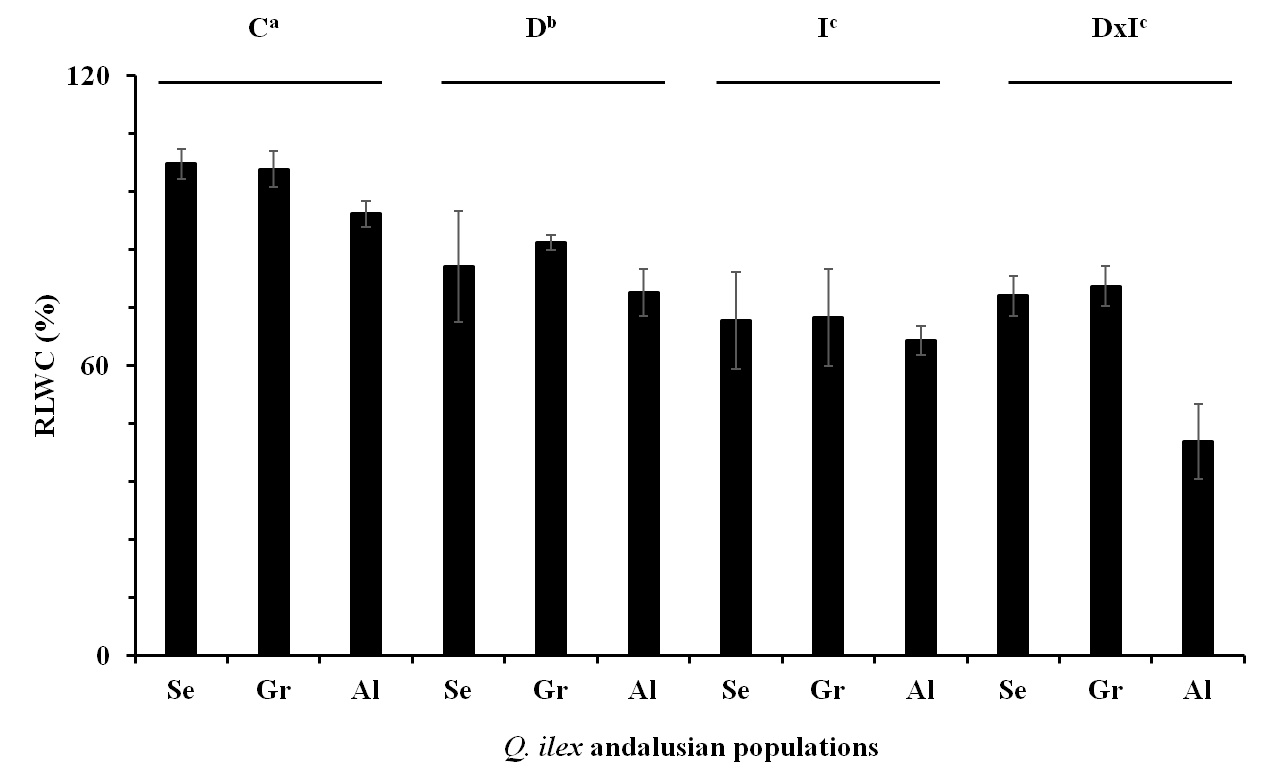


**Supplementary Figure 4|** **Relative leaf water content (RLWC, %) as determined on day 32 in seedlings from the Seville (Se), Granada (Gr) and Almeria (Al) populations.** Values are mean ± SE for three biological replicates. Statistically significant differences among treatments (C control; D drought; I inoculation; I; D×I combined) were observed. Different letters denote significant differences among treatments (*p* = 0.0000).


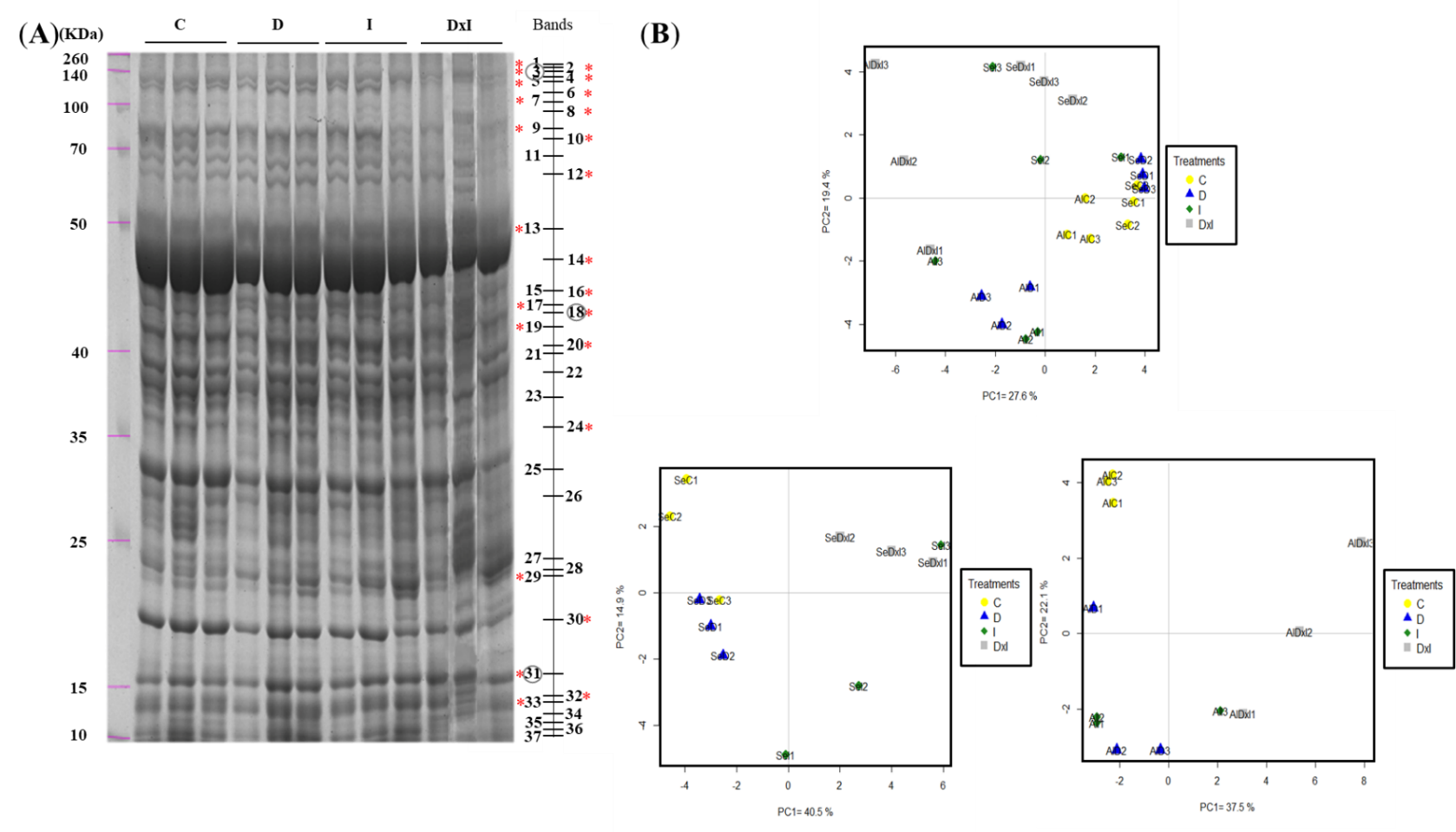


**Supplementary Figure 5| Protein profiles for leaves in seedlings from the Seville (Se) and Almeria (Al) populations as determined by 1-D gel electrophoresis (A).** The molecular marker (KDa) is shown on the left of the gel. Principal Component Analysis of all bands identified by 1-D analysis in both Se and Al (top), Se only (bottom-left) and Al only (bottom-right) (**B**).


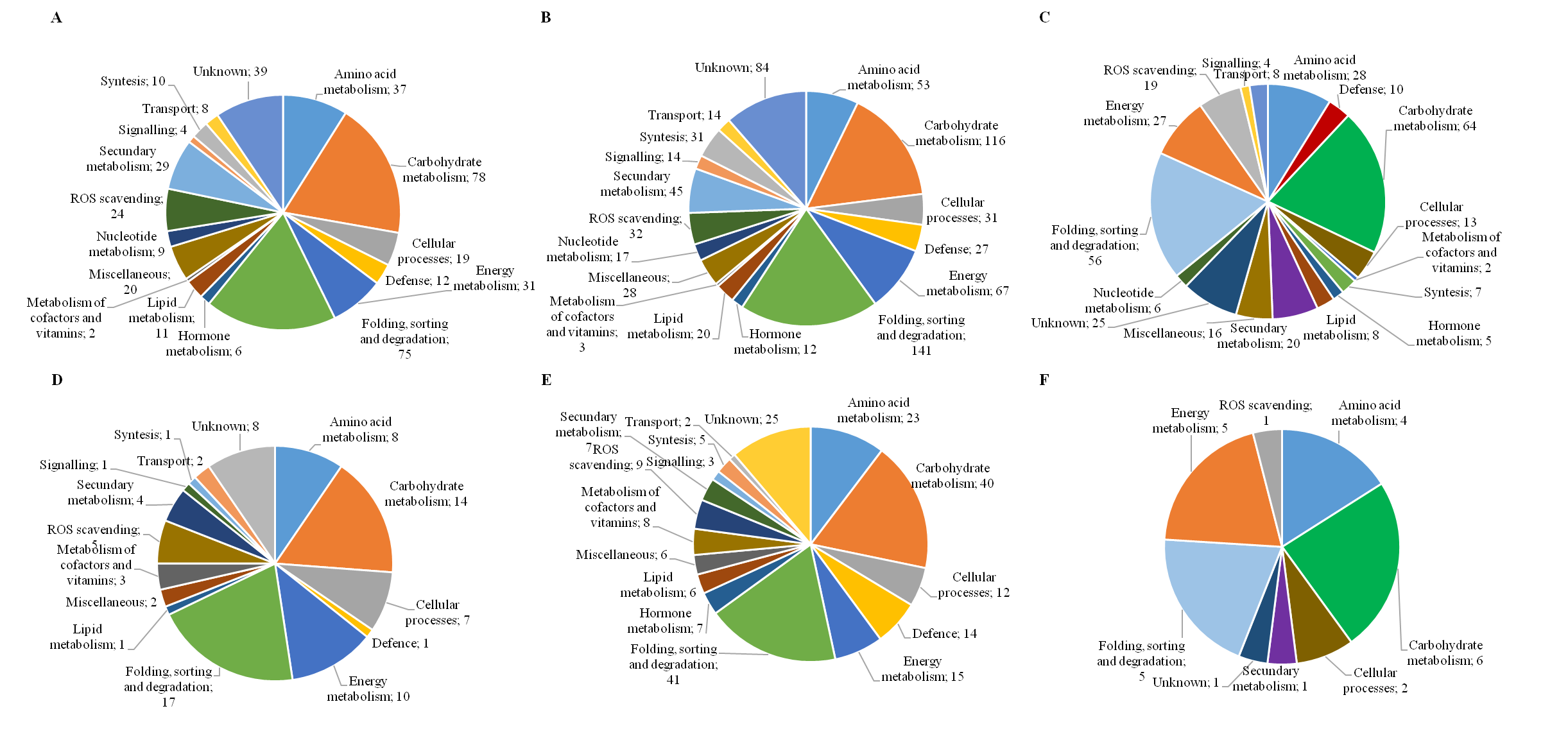


**Supplementary Figure 6| Datasets of total and variable proteins identified in Seville (A and D, respectively) and Almeria (B and E, respectively), and shared by the two populations (C and F, respectively), as grouped by molecular function with MERCATOR.** The total number of proteins in each category is shown.


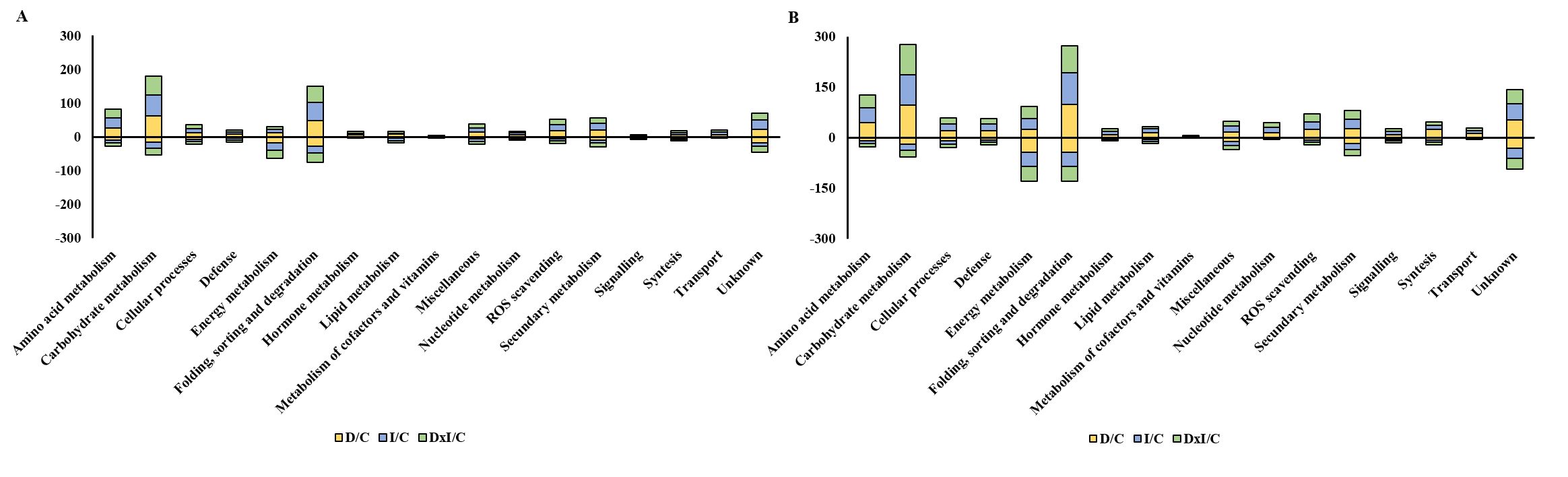


**Supplementary Figure 7| Number of up- and down-accumulated confidence proteins whose abundance in the drought (D), inoculated (I) and combined (D×I) treatments was greater or less than in the control treatment (C) in the Seville (A) and Almeria (B) populations.** The numbers of up- and down-accumulated proteins are represented by positive and negative values, respectively.


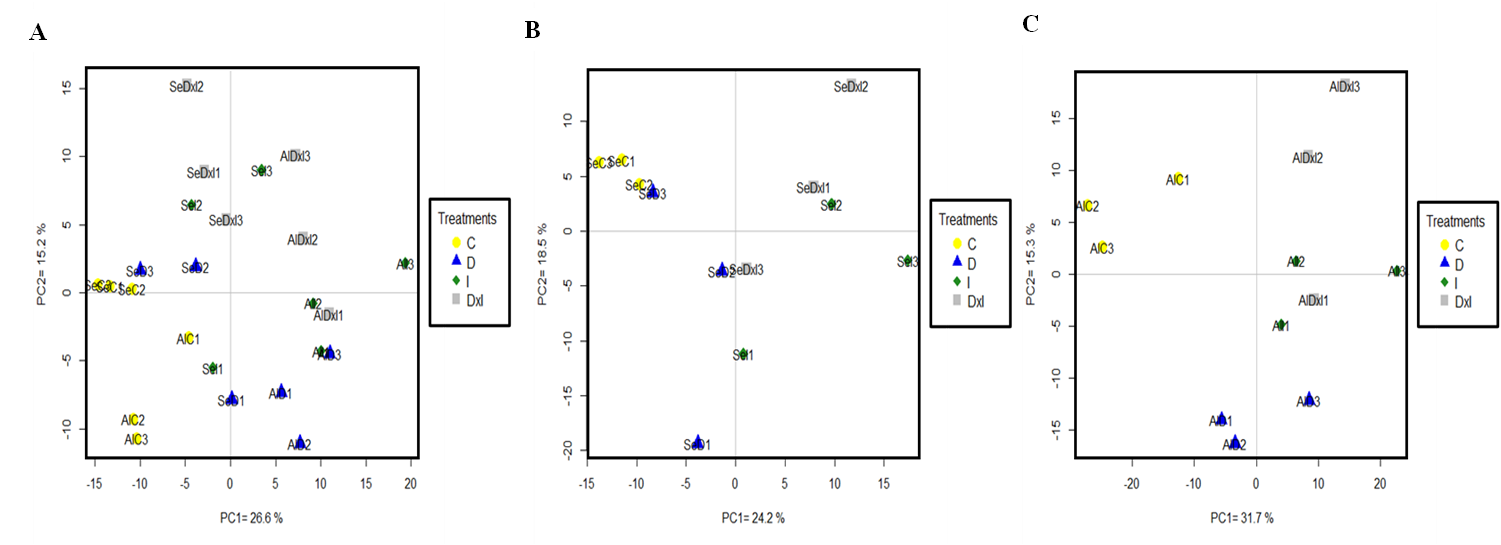
**Supplementary Figure 8| Principal Component Analysis of all confidence proteins identified in both populations (318, A), and only in Seville (Se) (414, B) or Almeria (Al) (734, C).**

**Supplementary Table S1| List of proteins identified by 1D (SDS-PAGE) and shotgun analysis in seedlings subjected to the control (C), drought (D), inoculation (I) and combined (D×I) treatment in the Seville (Se) and Almeria (Al) populations.** Different letters denote significant differences among treatments (*p* < 0.05).

Please refer a separate Excel file for **Supplementary Table S1**

**Supplementary Table S2| List by functional group of the first twenty proteins with positive and negative loadings in PC1 and PC2 in the Seville (Se) and Almeria (Al) populations.** C, D, I and D**×**I denote control, drought, inoculation and combined treatment, respectively.

| **Se population** |  |  |  |
| --- | --- | --- | --- |
| **Functional group** | **Protein** | **Up-accumulated** | **Loadings** |
| **PC1** |  |  |  |
| Amino acid metabolism | Cysteine synthase | I | 0,134671201 |
|  | Alanine--glyoxylate aminotransferase 2 homolog 1, mitochondrial | I; DxI | 0,138725675 |
| Carbohydrate metabolism | 6-phosphogluconate dehydrogenase, decarboxylating | I; DxI | 0,138255713 |
|  | Citrate synthase | I | 0,146813054 |
|  | Aldehyde dehydrogenase, mitochondrial | I; DxI | 0,150395963 |
| Cellular processes | Putative reversibly glycosylatable polypeptide | I | 0,145130599 |
|  | Alpha-1,4-glucan-protein synthase [UDP-forming] 2 | I; DxI | 0,148753876 |
| Folding, sorting and degradation | Proline iminopeptidase | I | 0,128417337 |
|  | Eukaryotic translation initiation factor 3 subunit F | I | 0,133480073 |
|  | ATP-dependent Clp protease proteolytic subunit-related protein 1, chloroplastic | I | 0,138556079 |
|  | 40S ribosomal protein S5 | I | 0,139542434 |
|  | Translocase of chloroplast | DxI | 0,145884352 |
| ROS scavending | FrnE protein-like | I; C | 0,133528064 |
| Secundary metabolism | NADPH-dependent codeinone reductase | DxI | 0,141369245 |
|  | NADPH:protochlorophyllide oxidoreductase porA | I; DxI | 0,142549926 |
| Signalling | Trans-2-enoyl-CoA reductase, mitochondrial | I; DxI | 0,130723221 |
| Transport | V-type proton ATPase subunit C | I | 0,143391600 |
| Unknown | Outer envelope pore protein 37, chloroplastic | I | 0,134589518 |
|  | BnaA01g04430D protein | I; DxI | 0,138201036 |
|  | Carboxylate clamp-tetratricopeptide repeat protein | I; DxI | 0,138579354 |
| Amino acid metabolism | Alpha isopropylmalate synthase | C | -0,122852515 |
|  | DS12 from 2D-PAGE of leaf protein, putative | C | -0,118663609 |
| Carbohydrate metabolism | Fructokinase | D | -0,090091392 |
| Cellular processes | Cell division protein FtsZ | C | -0,115483214 |
| Energy metabolism | 33kDa oxygen evolving protein of photosystem II | C | -0,134622801 |
|  | Post-illumination chlorophyll fluorescence increase protein | C | -0,126020067 |
|  | Phosphoglycolate phosphatase 1A, chloroplastic | C; D | -0,125797533 |
|  | Sedoheptulose-1,7-bisphosphatase | C; D | -0,119315646 |
|  | Ferredoxin--NADP reductase | C | -0,115981200 |
|  | Photosystem II subunit P-1 | C | -0,111722362 |
|  | Putative triosephosphate isomerase | D | -0,107149579 |
| Folding, sorting and degradation | HtrA-like protein | C; D | -0,138145231 |
|  | Peptidyl-prolyl cis-trans isomerase | C | -0,116390976 |
| Lipid metabolism | 3-oxoacyl-[acyl-carrier-protein] reductase, chloroplastic | C; D | -0,143973693 |
|  | Delta(3,5)-Delta(2,4)-dienoyl-CoA isomerase, peroxisomal | C; D; DxI | -0,097117324 |
| Metabolism of cofactors and vitamins | Pyridoxal 5'-phosphate synthase subunit PDX1.3 | D | -0,095867000 |
| Miscellaneous | Short-chain dehydrogenase TIC 32, chloroplastic | C | -0,111794987 |
| ROS scavending | Chloroplastic drought-induced stress protein of 32 KDa | C | -0,145548106 |
| Secundary metabolism | Glutamate synthase (Ferredoxin) | C | -0,088592860 |
| Unknown | Metallo-beta-lactamase domain-containing protein | D | -0,101354868 |
| **PC2** |  |  |  |
| Amino acid metabolism | Glyoxalase I | C; D; I | 0,203639693 |
|  | Arginine biosynthesis bifunctional protein ArgJ | I | 0,204108147 |
| Carbohydrate metabolism | Fructokinase | I | 0,120840694 |
|  | Alpha-amylase | I | 0,134587804 |
|  | HMG aldolase | C; D; I | 0,191073158 |
| Cellular processes | Cell division protein FtsZ | C | 0,121972298 |
|  | Patatin | I | 0,130841894 |
|  | Cell division protein FtsZ homolog 1, chloroplastic | C; D; I | 0,153978976 |
|  | Harpin binding protein 1 | C; D; I | 0,215407463 |
| Energy metabolism | Photosystem II subunit P-1 | C | 0,114099595 |
| Folding, sorting and degradation | Translocase of chloroplast | I | 0,136243712 |
|  | Mitochondrial processing peptidase | I | 0,163693915 |
|  | Mitochondrial-processing peptidase subunit beta | I | 0,170347898 |
|  | Peptidyl-prolyl cis-trans isomerase | C; D; I | 0,173743165 |
|  | Clone PI4869 proteasome inhibitor-like protein mRNA | I | 0,214512684 |
| Miscellaneous | Oxidoreductase, putative | I | 0,200450416 |
| Secundary metabolism | Caffeic acid O-methyltransferase | I | 0,121922782 |
| Unknown | Cysteine synthase | I | 0,11939092 |
|  | Fructose-bisphosphate aldolase | I | 0,129593872 |
|  | Lysine 6-aminotransferase | I | 0,173751918 |
| Amino acid metabolism | Glutamate decarboxylase | DxI | -0,147065708 |
|  | Alanine--glyoxylate aminotransferase 2 homolog 1, mitochondrial | I; DxI | -0,076726628 |
| Carbohydrate metabolism | Glucose-6-phosphate isomerase 1, chloroplastic | DxI | -0,171260663 |
|  | ATP-citrate synthase | I; DxI | -0,082207174 |
|  | 6-phosphogluconate dehydrogenase, decarboxylating | I; DxI | -0,063926424 |
|  | Succinate-semialdehyde dehydrogenase | D | -0,042583236 |
| Cellular processes | UDP-glucose 6-dehydrogenase 2 | DxI | -0,127661991 |
|  | Alpha-1,4-glucan-protein synthase [UDP-forming] 2 | I; DxI | -0,039356982 |
| Energy metabolism | Ferredoxin--NADP reductase | C | -0,133181025 |
|  | Post-illumination chlorophyll fluorescence increase protein | C | -0,073073096 |
| Folding, sorting and degradation | Haloacid dehalogenase-like hydrolase domain-containing protein At4g39970 | DxI | -0,180509786 |
|  | 50S ribosomal protein L5, chloroplastic | DxI | -0,148120852 |
|  | 40S ribosomal protein S5 | DxI | -0,130680831 |
| Lipid metabolism | Delta(3,5)-Delta(2,4)-dienoyl-CoA isomerase, peroxisomal | C; D; DxI | -0,070512505 |
| ROS scavending | Ascorbate peroxidase | DxI | -0,089730641 |
|  | L-ascorbate peroxidase, cytosolic | D; DxI | -0,080329601 |
|  | Thylakoid ascorbate peroxidases | I; DxI | -0,066094269 |
|  | Chloroplastic drought-induced stress protein of 32 KDa | C | -0,052175967 |
| Secundary metabolism | NADPH-dependent codeinone reductase | DxI | -0,081065206 |
| Transport | Putative beta-subunit of K+ channels | DxI | -0,134098229 |
| **Al population** |  |  |  |
| **Functional group** | **Protein** | **Up-accumulated** | **Loadings** |
| **PC1** |  |  |  |
| Carbohydrate metabolism | Glucose-6-phosphate 1-dehydrogenase, cytoplasmic isoform 2 | DxI | 0,083620512 |
|  | Aldehyde dehydrogenase | DxI; I; D | 0,083884994 |
|  | Serine hydroxymethyltransferase | DxI | 0,083941959 |
|  | Malic enzyme | DxI; I | 0,084150059 |
|  | NADH dehydrogenase [ubiquinone] flavoprotein 2, mitochondrial | DxI; I; D | 0,084854012 |
|  | ATP synthase subunit alpha, mitochondrial | DxI; I; D | 0,085624630 |
|  | Formate dehydrogenase | DxI; I; D | 0,085695392 |
|  | Malic enzyme | DxI; I | 0,086636987 |
|  | Glucose-1-phosphate adenylyltransferase large subunit, chloroplastic/amyloplastic | I | 0,086639036 |
|  | Malate dehydrogenase | DxI; I; D | 0,088034545 |
| Cellular processes | UDP-D-apiose/UDP-D-xylose synthase | I | 0,084723943 |
|  | Alpha-1,4-glucan-protein synthase [UDP-forming] 2 | DxI; I; D | 0,086561347 |
| Energy metabolism | Fructose-bisphosphate aldolase | DxI; I; D | 0,086927615 |
| Folding, sorting and degradation | Heat shock protein 60 | DxI; I; D | 0,085004175 |
|  | T-complex protein 1 subunit zeta 1 | DxI; I; D | 0,089137549 |
| Hormone metabolism | Aluminum induced protein with YGL and LRDR motifs | DxI; I; D | 0,084157903 |
|  | Probable aldo-keto reductase 1 | DxI; I; D | 0,085397904 |
| Lipid metabolism | Acyl-[acyl-carrier-protein] desaturase | I | 0,087659507 |
| Miscellaneous | Dynamin-related protein 1E | DxI; I | 0,086364345 |
| ROS scavending | Monothiol glutaredoxin-S17 | DxI; I; D | 0,083444644 |
| Carbohydrate metabolism | Granule-bound starch synthase 1, chloroplastic/amyloplastic | C | -0,084957955 |
| Cellular processes | Cell division protein FtsZ | C | -0,082806716 |
|  | Cell division protein FtsZ | C | -0,078876747 |
|  | Chloroplast FtsZ-like protein | C | -0,079957562 |
| Energy metabolism | Photosystem II subunit P-1 | C | -0,088594741 |
|  | PsbP domain-containing protein 3, chloroplastic | C | -0,083583344 |
| Folding, sorting and degradation | HtrA-like protein | C | -0,087566713 |
|  | Probable ADP-ribosylation factor GTPase-activating protein AGD8 | C | -0,080434393 |
|  | ATP-dependent zinc metalloprotease FTSH, chloroplastic | C | -0,078158888 |
| Miscellaneous | Short-chain dehydrogenase TIC 32, chloroplastic | C | -0,077959055 |
| ROS scavending | Ferredoxin-thioredoxin reductase, variable chain | C | -0,085498846 |
| Secundary metabolism | 2OG-Fe(II) oxygenase family oxidoreductase | C | -0,086286533 |
| Syntesis | BnaA04g05670D protein | C | -0,080866612 |
| Unknown | Thylakoid lumenal 19 kDa protein, chloroplastic | C | -0,087292919 |
|  | BnaC06g35550D protein | C | -0,086218024 |
|  | AT5g48790/K24G6_12 | C | -0,08544842 |
|  | Thylakoid lumenal protein TL20.3, chloroplastic | C | -0,083354229 |
|  | NmrA family protein | C | -0,083036501 |
|  | Putative uncharacterized protein Sb01g001480 | C | -0,079180102 |
|  | BnaAnng11920D protein | C | -0,078428047 |
| **PC2** |  |  |  |
| Amino acid metabolism | Ornithine carbamoyltransferase, chloroplastic | I; DxI | 0,067037801 |
|  | Methionine synthase | DxI | 0,086406313 |
| Carbohydrate metabolism | Aldehyde dehydrogenase, mitochondrial | DxI | 0,069051721 |
|  | Glucose-6-phosphate isomerase 1, chloroplastic | DxI | 0,089556889 |
|  | Serine hydroxymethyltransferase | DxI | 0,094407201 |
|  | Carbonate dehydratase | DxI | 0,105989354 |
| Defense | BURP domain protein RD22 | DxI | 0,086388976 |
| Energy metabolism | Ferredoxin--NADP reductase | DxI | 0,102371216 |
|  | Serine--glyoxylate aminotransferase | DxI | 0,123546721 |
| Folding, sorting and degradation | 40S ribosomal protein S19 | DxI | 0,068660324 |
|  | AMPP | DxI | 0,075187168 |
|  | 50S ribosomal protein L5, chloroplastic | DxI | 0,087674819 |
|  | 60S ribosomal protein L17-2 | DxI | 0,104318614 |
| Hormone metabolism | 12-oxophytodienoate reductase 1 | I; DxI | 0,068398959 |
| Miscellaneous | CTF2A like oxidoreductase | DxI | 0,098176129 |
| Nucleotide metabolism | Adenylate kinase | I; DxI | 0,102221691 |
| ROS scavending | Catalase | I; DxI | 0,109176303 |
|  | Catalase | I; DxI | 0,118027832 |
| Secundary metabolism | Aminotransferase | I; DxI | 0,073624373 |
| Signalling | Sulfite reductase 1 [ferredoxin], chloroplastic | DxI | 0,099056469 |
| Amino acid metabolism | Imidazole glycerol phosphate synthase hisHF, chloroplastic | D | -0,122384852 |
| Carbohydrate metabolism | ATP synthase subunit beta, mitochondrial | D | -0,136418545 |
|  | Fructokinase | D | -0,126751979 |
|  | Enolase | D | -0,120874414 |
|  | Bifunctional protein FolD 4, chloroplastic | D | -0,118898426 |
| Cellular processes | Multiple organellar RNA editing factor 2, chloroplastic | D | -0,131707619 |
|  | PPIase | D; I | -0,122491011 |
| Defense | DnaK | D | -0,136292566 |
|  | Putative chaperone protein ClpB2, chloroplastic | D | -0,132483917 |
|  | Heat shock cognate 70 kDa protein | D | -0,124403571 |
| Folding, sorting and degradation | Proteasome subunit beta type-7-A | D | -0,134978520 |
|  | Ubiquitin-conjugating enzyme E2 variant 1D | C; D; I | -0,118637713 |
|  | Elongation factor Tu, mitochondrial | D; I | -0,112276626 |
| Lipid metabolism | Dihydrolipoyllysine-residue acetyltransferase component 4 of pyruvate dehydrogenase complex, chloroplastic | D | -0,132368462 |
| Folding, sorting and degradation | PSA6 | D | -0,140688805 |
| ROS scavending | Ascorbate peroxidase | D | -0,121202464 |
| Signalling | Ran-specific GTPase-activating protein | D | -0,142335340 |
| Transport | Vacuolar H(+)-ATPase | D | -0,113504727 |
| Unknown | Uncharacterized protein At2g27730, mitochondrial | D | -0,131049393 |
|  | Major allergen Pru ar, putative | D | -0,128274176 |
